# Transcriptomic analysis of crustacean molting gland (Y-organ) regulation via the mTOR signaling pathway

**DOI:** 10.1038/s41598-018-25368-x

**Published:** 2018-05-09

**Authors:** S. Shyamal, S. Das, A. Guruacharya, D. L. Mykles, D. S. Durica

**Affiliations:** 1 Department of Biology, University of Oklahoma, Norman, Oklahoma 73019 USA; 2 Department of Biology, Colorado State University, Fort Collins, Colorado, 80523 USA

## Abstract

The intermolt crustacean Y-organ (YO) maintains a basal state mediated by pulsatile release of molt inhibiting hormone (MIH), a neuropeptide produced in the eyestalk ganglia, inhibiting YO ecdysteroidogenesis. Reduction of MIH results in YO activation and the animal enters premolt. In the crab, *Gecarcinus lateralis*, molting was induced by eyestalk ablation (ESA). ESA animals were injected with either rapamycin, an mTOR inhibitor, or DMSO vehicle at Day 0. YOs were harvested at 1, 3, and 7 days post-ESA and processed for high throughput RNA sequencing. ESA-induced increases in mRNA levels of mTOR signaling genes (e.g., *mTOR*, *Rheb*, *TSC1*/*2*, *Raptor*, *Akt*, and *S6 kinase*) declined following rapamycin treatment. In concert with mTOR inhibition, mRNA levels of ecdysteroid biosynthesis genes (e.g., *Nvd*, *Spo*, *Sad*, *Dib*, and *Phm*) were decreased and accompanied by a decrease in hemolymph ecdysteroid titer. By contrast, rapamycin increased the mRNA level of *FKBP12*, the rapamycin-binding protein, as well as the mRNA levels of genes associated with Wnt and insulin-like growth factor signaling pathways. Many MIH and transforming growth factor-β signaling genes were down regulated in ESA animals. These results indicate that mTOR activity either directly or indirectly controls transcription of genes that drive activation of the YO.

## Introduction

The Y-organ (YO), or molting gland, is the source of steroid hormone production and consequent molt cycle regulation in decapod crustaceans, and is responsive to both external environmental and internal physiological signals^1–3^. Control of molting involves a complex interaction between the eyestalk neurosecretory center, which produces inhibitory neuropeptides, such as molt-inhibiting hormone (MIH) and crustacean hyperglycemic hormone (CHH), and the paired YOs in the anterior cephalothorax^2,4–6^. The YO undergoes transitions in physiological properties at critical stages of the molt cycle. During intermolt, the YO is kept in the basal state by pulsatile releases of MIH to maintain low hemolymph ecdysteroid titers^7,8^. A reduction in circulating MIH relieves YO repression. The activated YO hypertrophies to increase ecdysteroid synthesis; the hemolymph ecdysteroid titer increases and the animal transitions to the premolt stage^1,5^. Autotomy, the reflexive loss of a limb due to injury, suspends premolt for a few weeks, which allows time for a new regenerate to form and grow, and the animal molts with a complete set of walking legs^5,9,10^. A critical transition occurs at mid-premolt, when the animal becomes committed to molt. The committed YO increases ecdysteroid production further and becomes insensitive to MIH and CHH and a regulatory signal associated with the regenerating limb^5,10,11^. The animal progresses through to ecdysis without delay.

**Figure 1:**
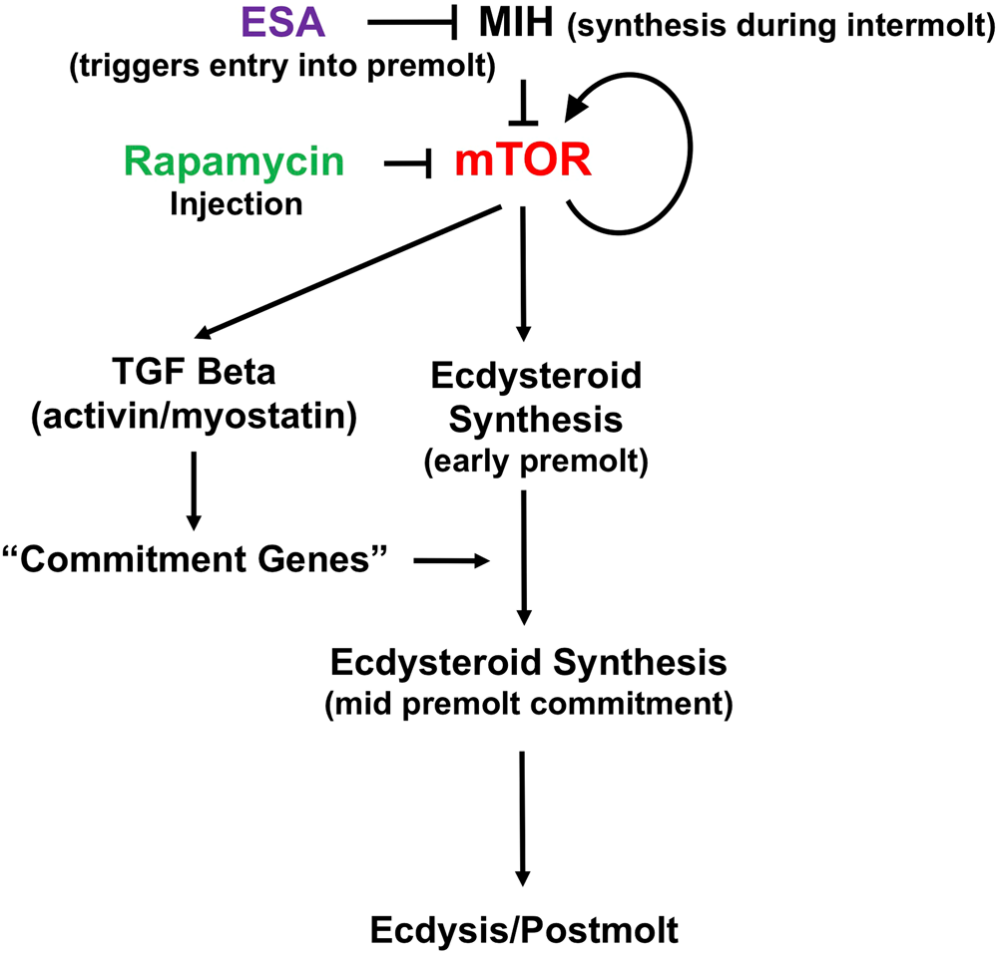
Model outlining regulatory pathways affecting progression through molt. During intermolt, production of eyestalk MIH neuropeptide inhibits mTOR signaling and YO ecdysteroid synthesis. Eyestalk ablation triggers entry into premolt and ecdysteroid production via mTOR signaling activation. mTOR signaling invokes TGFβ signaling, leading to progression into mid- premolt, YO commitment through mid and late premolt, sustained ecdysteroidogenesis and concomitant ecdysis. mTOR inhibition via rapamycin injection depresses ecdysteroid synthesis and feeds back on mTOR and TGFβ signaling pathways.

Several gene regulatory pathways have been implicated in controlling the transition into the premolt state and maintenance of ecdysteroid production (Fig. 1). It is hypothesized that the MIH signaling pathway consists of a cAMP-dependent triggering phase and an NO/cGMP-dependent summation phase, which maintains the YO in the basal state between MIH pulses^4,5,11,12^. YO activation in early premolt requires mechanistic target of rapamycin (mTOR)-dependent protein synthesis, as rapamycin, an mTOR antagonist, inhibits *G*. *lateralis* YO ecdysteroid secretion *in vitro*^13^ and ESA-induced increases in hemolymph ecdysteroid titer *in vivo*^14^. In insects, mTOR is required for ecdysteroidogenesis in the prothoracic gland^11^. mTOR is a phosphoinositide 3-kinase (PI3K)-related kinase that controls gene expression at both the transcriptional and translational levels in eukaryotic cells^15–17^. MTOR can form two multi-protein complexes, mTORC1 and mTORC2, that through kinase-mediated phosphorylation regulate cell growth, proliferation, and survival^18,19^. mTORC1 is directly regulated by Rheb (Ras homolog expressed in brain). Rheb/GTP activates mTORC1 and is inactivated by Rheb-GTPase activating protein (Rheb-GAP or TSC1/2). TSC1/2 is in turn inhibited by phosphorylation by Akt^20^. mTOR acts downstream of and feeds back on Akt, which operates at a key junction in the PI3K pathway^21,22^. In addition to translational control, increases in *G*. *lateralis* mRNA levels of mTOR signaling components *Gl*-*mTOR* and *Gl-Akt* coincide with increased YO ecdysteroid production during mid and late premolt stages^13^. Eyestalk ablation (ESA) increases mRNA levels of *Gl-mTOR*, *Gl-Akt*, and *Gl-S6 kinase* (*S6K*)^14^.

TGFβ/Smad signaling via an activin-like membrane receptor has been implicated in sustained ecdysteroid production and hypothesized to mediate the transition of the YO from the activated to the committed state^5^. Quantitative PCR studies indicate ESA increases *Gl-Myostatin* (*Gl-Mstn*) expression in the YO^14^. Injection of SB431542, an activin receptor antagonist, in ES-ablated *G*. *lateralis* blocks the increase in ecdysteroid titer associated with the transition to YO commitment^14^. Moreover, SB431542 lowers mRNA levels of *Gl-mTOR*, *Gl-Rheb*, *Gl-Akt*, *Gl-S6K*, and *Gl-EF2* in ES-ablated *G*. *lateralis*^14^. In the ecdysteroidogenic *Drosophila* prothoracic gland, knockdown of the Activin/Babo pathway genes causes developmental arrest before metamorphosis through the regulation of prothoracicotropic hormone (PTTH) and insulin-like peptides (ILPs)^23–26^. TGFβ signaling in insects interfaces with the regulation of both ecdysteroid (20-hydroxyecdysone or 20E) and sequiterpenoid (juvenile hormone) synthesis during growth and nutritional consumption^25,27^. We hypothesize that Gl-Mstn may have a similar function in crustaceans, as the YO shows decreased ecdysteroid production when TGFβ signaling is inhibited^14^ and shows a continuous increase in ecdysteroid synthesis in response to reduced sensitivity to MIH^5,7^. Taken together, these data suggest that mTOR and TGFβ/Smad pathways play essential roles in the regulation of both crustacean and insect molting glands by neuropeptides.

Given the diversity of species and paucity of information on genome structure, there is a need for broader application of bioinformatic and statistical tools towards gene identification and gene expression quantification in Pancrustacea. Our ability to detect adaptive shifts and to address mechanistic questions regarding phenotypic plasticity in non-model organisms, especially crustaceans, is limited. The advent of next generation sequencing technologies has given us the ability to examine whole transcriptomes, and explore changes in gene expression relative to changes in native physiological state or experimental intervention/manipulation^28^. MIH, mTOR, and TGFβ signaling pathways are among 23 signal transduction pathways expressed in the *G*. *lateralis* YO transcriptome^29^. The present study examines global changes in YO gene expression on entering the molt cycle, and the effects of mTOR inhibition on gene pathways involved in YO ecdysteroidogenesis. ESA was used to induce molting in *G*. *lateralis*. Illumina high-throughput sequencing, and bioinformatics analyses permitting *de novo* assembly, gene annotation, and quantification of gene expression were used to characterize ESA-induced changes in the YO transcriptome. In addition, the effects of inhibiting mTOR signaling via rapamycin injection were examined. Although the mTOR pathway has been associated with regulation at the translational level, this analysis indicates mTOR pathway interference contributes to both increases and decreases in gene transcription associated with genes encoding putative regulatory pathway components, as well as the down-regulation of ecdysteroid biosynthetic pathway genes.

## Results

### Sequencing and assembly of the YO transcriptome

YO transcriptome libraries were constructed from intact, intermolt animals (I library; stage C_4_) and ES-ablated animals transitioning from intermolt into premolt as a consequence of acute MIH withdrawal. Both DMSO-injected control libraries (D libraries) and rapamycin-injected experimental libraries (R libraries) were produced at three time points following injection (Day 1 = A; Day 3 = C; Day 7 = G). Three YO transcriptome technical replicates (1/2/3) were generated for each time point (designated I-1/2/3; DA-1/2/3, DC-1/2/3; DG-1/2/3; RA-1/2/3/; RC-1/2/3; and RG-1/2/3). Three animals, representing 6 paired YOs, were used for each replicate. The raw data for the transcriptomic analysis was collected from Illumina HiSeq 2000 sequencer reads and subsequently assembled to generate the comparator intact/intermolt (I) and individual D and R premolt transcriptome libraries.

**Figure 2:**
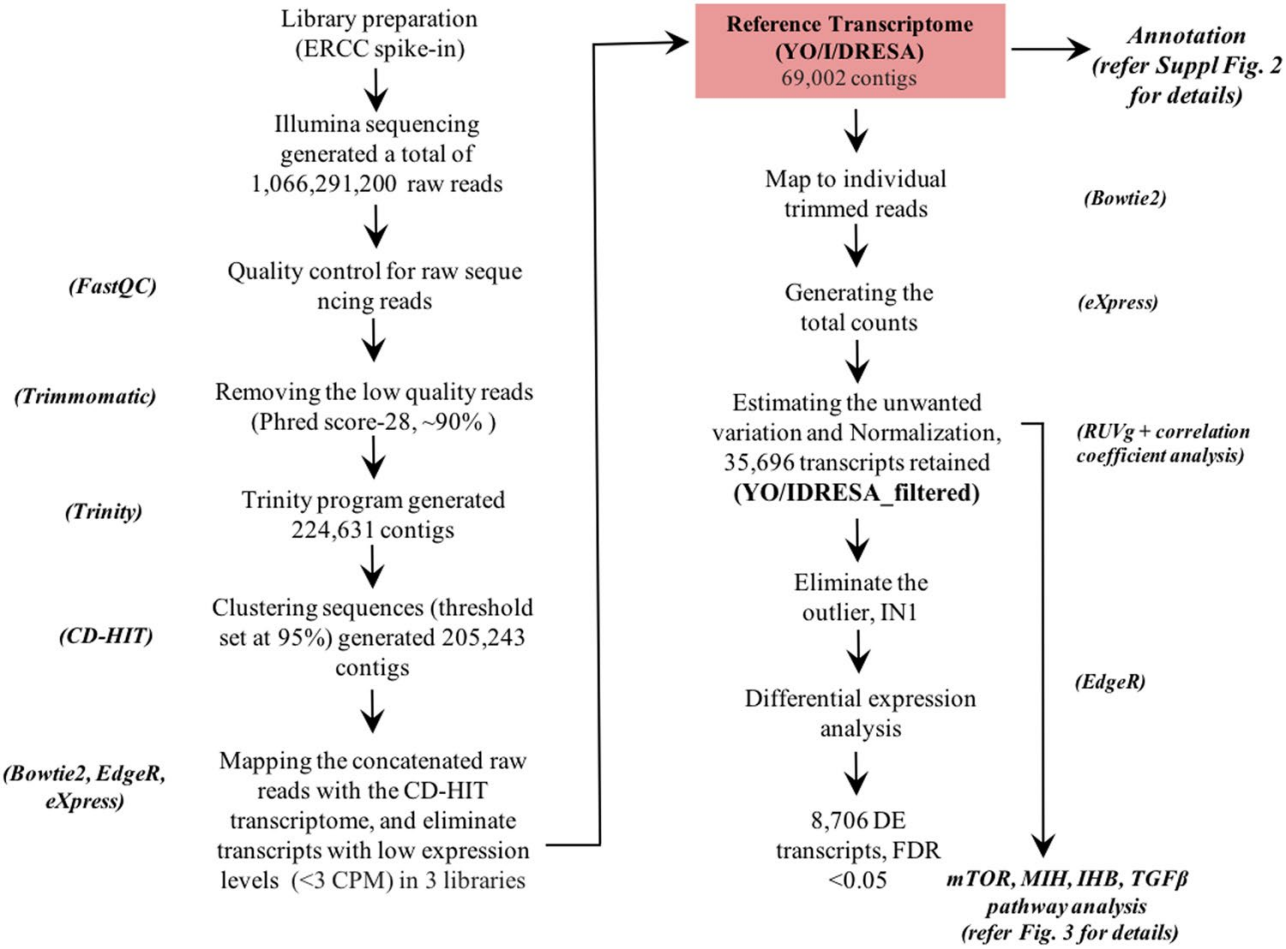
*De novo* assembly pipeline for *G*. *lateralis* YO/IDRESA transcriptome. The software required for assembling raw reads from Illumina sequencing, data evaluation, *de novo* assembly, estimation of transcript abundance, and contig annotation are indicated in italics. A detailed description of the software pipeline is provided in the Methods section and Supplementary scripts.

A *G*. *lateralis* genome is not available and *de novo* transcriptome assembly was initiated using several integrated software packages (Fig. 2). Table 1 provides library comparisons of quality reads recovered after trimming; 89–92% of the reads mapped back to the reference transcriptome. Illumina sequencing generated a total of 1,066,291,200 raw reads (Fig. 2). Following trimming (Phred quality score-28), ~90% of the reads were retained for further analysis. High quality reads were assembled via the Trinity program^30^, which generated 224,631 contigs (Fig. 2 and Table 2). To reduce the number of redundant sequences, the CD-HIT-EST program was used to cluster similar sequences at a threshold set at 95% nucleotide similarity^31^. The output from the CD-HIT-EST program (205,243 contigs) was used for filtering the dataset.

**Table 1:** Raw reads, reads following trimming and reads mapped back to transcriptome (RMBT) for three technical replicates of *G*. *lateralis* intact/intermolt (I), ESA control (D), and rapamycin treated (R) libraries.

Library	Total raw reads from Illumina	Quality reads following trimming	RMBT following mapping via Bowtie2
I1	56,495,577	45,117,841	92.19%
I2	64,413,406	48,305,338	91.95%
I3	64,056,380	48,229,308	91.71%
DA1	62,273,583	48,747,261	92.47%
DA2	50,268,578	38,913,595	90.23%
DA3	62,657,503	46,994,892	91.78%
DC1	46,731,225	36,723,723	90.54%
DC2	56,292,057	43,996,686	89.30%
DC3	39,961,197	30,506,863	88.06%
DG1	47,870,500	37,322,171	88.82%
DG2	46,684,320	34,014,057	91.58%
DG3	38,754,359	29,407,917	90.34%
RA1	62,419,293	47,390,266	93.10%
RA2	43,477,315	33,107,576	93.19%
RA3	51,417,450	39,803,322	90.82%
RC1	49,670,451	36,328,424	89.45%
RC2	25,264,893	25,059,946	91.41%
RC3	55,304,730	43,221,116	90.76%
RG1	48,662,749	36,440,457	91.42%
RG2	51,141,698	37,975,656	89.96%
RG3	42,473,936	32,704,772	90.38%

**Table 2:** Characterization of transcriptome assembly via Trinity.

Trinity Assignments	Number of contigs with hits or assignments
Total Illumina raw reads	1,066,291,200
Total number Trinity assembled contigs	224,631
Number of clusters (95%-CD-HIT-EST output)	205,243
Filtering using EdgeR to eliminate low expression contigs (reference transcriptome)	69,002 (33.61%)
Total trinity ‘genes’	56,110
Total trinity transcripts (-ERCC spike-ins)	68,929
Median contig length in bp	940
Contig (N50 in bp)	1,997
Average contig in bp	1,364
Number of predicted peptides (Transdecoder)	66,167
Transcripts after RUVg filtering (filtered_transcriptome)	35,696 (51.73%)
Differentially expressed (DE)	8,706 (24.38%)

### Filtering, estimating factors of unwanted variation via RUVg normalization

Bowtie2 was used to map the reads from individual libraries back to the CD-HIT assembled transcriptome^32^. Contigs with very low counts across all libraries were removed by using EdgeR filtering (filtering criteria given in Section 4.4, <3 CPM in replicate libraries)^33^. EdgeR filtering ensured that genes were retained when consistently expressed in all replicate libraries. EdgeR filtering retained 33.61% (69,002) of the total CD-HIT assembled transcriptome (Table 2). This was designated as the reference transcriptome (YO/I/DRESA) (Fig. 2 and Table 2). Statistics characterizing the reference transcriptome assembly process are summarized in Table 2. Filtering the Trinity transcripts produced 69,002 contigs with a median length of 940 bp, an average contig length of 1,364 bp, and an N50 contig length of 1,997 bp (Table 2). The contig length values for the YO/I/DRESA transcriptome assembly in our study (Table 2) are comparable to other previously reported assembled crustacean *de novo* transcriptomes (e.g. 870–1435 bp average contig length)^29,34–36^. Read alignment was similar among the three replicates; 88% to 93% of the individual library reads mapped back to the reference transcriptome (Table 1).

RUVg software was used to improve normalization of RNA-seq data by estimating unwanted variation due to library preparation artifacts^37^. To this end, synthetic negative controls of various lengths and GC content were designed that would not be influenced by the biological covariates under study. We employed the External RNA Control Consortium (ERCC) assembly of 92 spike-in controls^37^ in the normalization procedure. RUVg analysis assessed two parameters: (1) the estimated factors of unwanted variation and (2) the normalized counts obtained by regression of the original counts on the unwanted factors. There were substantial library preparation effects for un-normalized counts (Suppl. Figure 1A,B). These were only attenuated (and not fully removed) by upper-quartile normalization (Suppl. Figure 1C,D). By contrast, RUVg reduced library preparation effects (Suppl. Figure 1E,F). After RUVg filtering, we were left with 35,696 genes (Table 2) and 42 ERCC spike-ins (Fig. 2). This dataset was designated as the filtered transcriptome (YO/I/DRESA_filtered). Both count and FPKM values (fragments per kilobase of contig per million fragments mapped) were positively correlated between replicates (P = 0.81–0.99), indicating that the normalization protocols controlled for potential library preparation artifacts and allowed for interlibrary comparisons (Table 3). If gene expression differences exist among experimental conditions, it is expected that biological replicates of the same condition will cluster together in a principal component analysis (PCA) (Suppl. Figure 1F). Pearson coefficients were calculated in a pairwise statistical analysis of count and FPKM values (Table 3). In general, the biological replicates clustered in the same treatments and times, with PC1 explaining 49.5% of the variation and PC2 explaining another 11.8% of the variation after normalization (Suppl. Figure 1F). In this analysis, one of the intermolt library replicates (I1) was observed as an outlier, as the correlation coefficient value was less than 0.6. Since this replicate remained an outlier following normalization (Suppl. Figure 1D,F and Table 3), it was excluded from differentially expressed gene analysis (DEG) of the dataset.

**Table 3:** Pearson correlation coefficients between the biological replicates.

	I1	I2	I3				
**Using count data**							
I1	1	***0***.***81***	***0***.***56***				
I2		1	0.76				
I3			1				
	**DA1**	**DA2**	**DA3**		**RA1**	**RA2**	**RA3**
DA1	1	0.95	0.99	**RA1**	1	0.97	0.98
DA2		1	0.96	**RA2**		1	0.97
DA3			1	**RA3**			1
	**DC1**	**DC2**	**DC3**		**RC1**	**RC2**	**RC3**
DC1	1	0.99	0.99	**RC1**	1	0.97	0.94
DC2		1	0.99	**RC2**		1	0.96
DC3			1	**RC3**			1
	**DG1**	**DG2**	**DG3**		**RG1**	**RG2**	**RG3**
DG1	1	0.98	0.98	**RG1**	1	0.95	0.96
DG2		1	0.94	**RG2**		1	0.94
DG3			1	**RG3**			1
**Using FPKM data**							
	**IN1**	**IN2**	**IN3**				
IN1	1	***0***.***59***	***0***.***34***				
IN2		1	*0*.*81*				
IN3			1				
	**DA1**	**DA2**	**DA3**		**RA1**	**RA2**	**RA3**
DA1	1	0.88	0.98	**RA1**	1	0.95	0.96
DA2		1	0.89	**RA2**		1	0.96
DA3			1	**RA3**			1
	**DC1**	**DC2**	**DC3**		**RC1**	**RC2**	**RC3**
DC1	1	0.95	0.95	**RC1**	1	0.93	0.78
DC2		1	0.96	**RC2**		1	0.88
DC3			1	**RC3**			1
	**DG1**	**DG2**	**DG3**		**RG1**	**RG2**	**RG3**
DG1	1	0.93	0.97	**RG1**	1	0.86	0.81
DG2		1	0.94	**RG2**		1	0.94
DG3			1	**RG3**			1

### Reference transcriptome annotation

Using the YO/I/DRESA_transcriptome output of 69,002 contigs, BLASTx analysis against the NCBI NR database produced 16,003 ‘hypothetical/predicted’ protein assignments. To facilitate the identification of transcripts with known genes, the BLASTx output (69,002 contigs) was analyzed against the Swiss-Prot (SP) and TrEMBL (http://www.uniprot.org/downloads) databases (Table 4). This resulted in 12,066 (17.48%) and 11,788 (17.08%) transcript gene assignments, respectively, using 1e–5 as the e-value cutoff (Table 4).

**Table 4:** Annotation summary for YO/IDR/ESA transcriptome.

Annotation mode or tool	Number of contigs with hits or assignments	Hits (%)	Number of contigs assigned to same accession
BLASTx against Swiss-Prot	12,066	17.48	2,372
BLASTx against TrEMBL-Uniprot	11,788	17.08	3,651
BLASTp against Swiss-Prot	10,993	16.61	3,095
BLASTp against TrEMBL-Uniprot	10,778	16.28	2,984

In addition to this direct CD-HIT-EST output search, open reading frames (ORFs) were generated from the CD-HIT-EST contig output using TransDecoder (Table 5). This resulted in 66,167 predicted peptides, of which 41,213 (62.3%) were complete (containing both start and stop codons), 14,999 (22.7%) were 5′-partial (missing a start codon), 5,420 (8.2%) were 3′-partial (missing a stop codon), and 4,535 (6.9%) were internal (lacking both a start and stop codon). These ORFs were also annotated via BLASTp against the SP and Uniprot-UniRef90 databases. These BLAST analyses resulted in a comparable level of transcript assignment: 10,993 (16.6%) and 10,778 (16.3%) predicted peptides/ORFs with significant hits, respectively (Table 4). In addition to the BLAST analyses, the ORF sequences were analyzed to identify predicted Pfam domains (HMMER/Pfam), transmembrane helices (TMHMM) and signal peptide cleavage sites (SignalP). All the outputs from BLASTx, BLASTp, HMMER/Pfam, TMHMM, and SignalP were combined into a single annotation file using Trinotate (Suppl. Figure 2 and Table 5). This assembly resulted in 9,695 transcripts with predicted Pfam domains, 6,167 transcripts with predicted transmembrane helices, 1,099 transcripts with a signal peptide cleavage site, and 5,292 transcripts with GO (Gene Ontology) annotations from Pfam-A hits.

**Table 5:** Identification of predicted protein sequences using TransDecoder.

Types of peptide sequence	Number of contigs
Number of predicted peptides (TransDecoder)	66,167
Complete (ORF with both start and stop codons)	41,213
5′ partial (ORF missing a start codon)	14,999
3′ partial (ORF missing the stop codon)	5,420
Internal (ORF with no start or stop codons)	4,535
**Annotation Source**	
Pfam domains	9,695
TMHMM	6,167
SignalP	1,099
GO annotation from Swiss-Prot/TrEMBL	5,292
KEGG annotation using KAAS	4,887

Using the TransDecoder BLASTp hits (Table 5), the GO Slim program (http://geneontology.org/page/go-slim-and-subset-guide) was run to assign GO categories. Of the 66,167 TransDecoder BLASTp hits, 5,292 transcripts were assigned to at least one of the three parent GO categories: 4,533 were assigned to molecular function (MF), 4,776 were assigned to cellular component (CC), and 4,883 to biological process (BP). The distribution of unique transcripts annotated to one or a combination of the parent GO categories is shown in Suppl. Figure 3. Of the 5,292 GO assignments within the reference transcriptome, 692 were assigned to unique GO terms, while most exhibited multiple characterized domains/properties/activities. Many of the transcripts were assigned to two or all three categories. For example, there was considerable overlap of transcripts assigned to the CC and BP categories, with 1,573 transcripts assigned only to CC and 1,743 transcripts assigned only to BP. One-third (3,049) of the transcripts were assigned to all three categories. Within the MF category, the most represented GO Slim terms were ion binding (GO:1990243) and catalytic activity (GO:0016887) (Suppl. Figure 4A). In the cellular component category, the most represented GO Slim terms were cell (GO:0008285), organelle (GO:0000381), organelle part (GO:0006122), membrane (GO:0003723), and macromolecular complex (GO:0032991) (Suppl. Figure 4B). Single organism process (GO:0044802) and biological regulation (GO:0065007) were the most highly represented terms in the biological processes category (Suppl. Figure 4C).

### Pathway annotation: KEGG and comparative analysis of the filtered reference transcriptome

**Figure 3:**
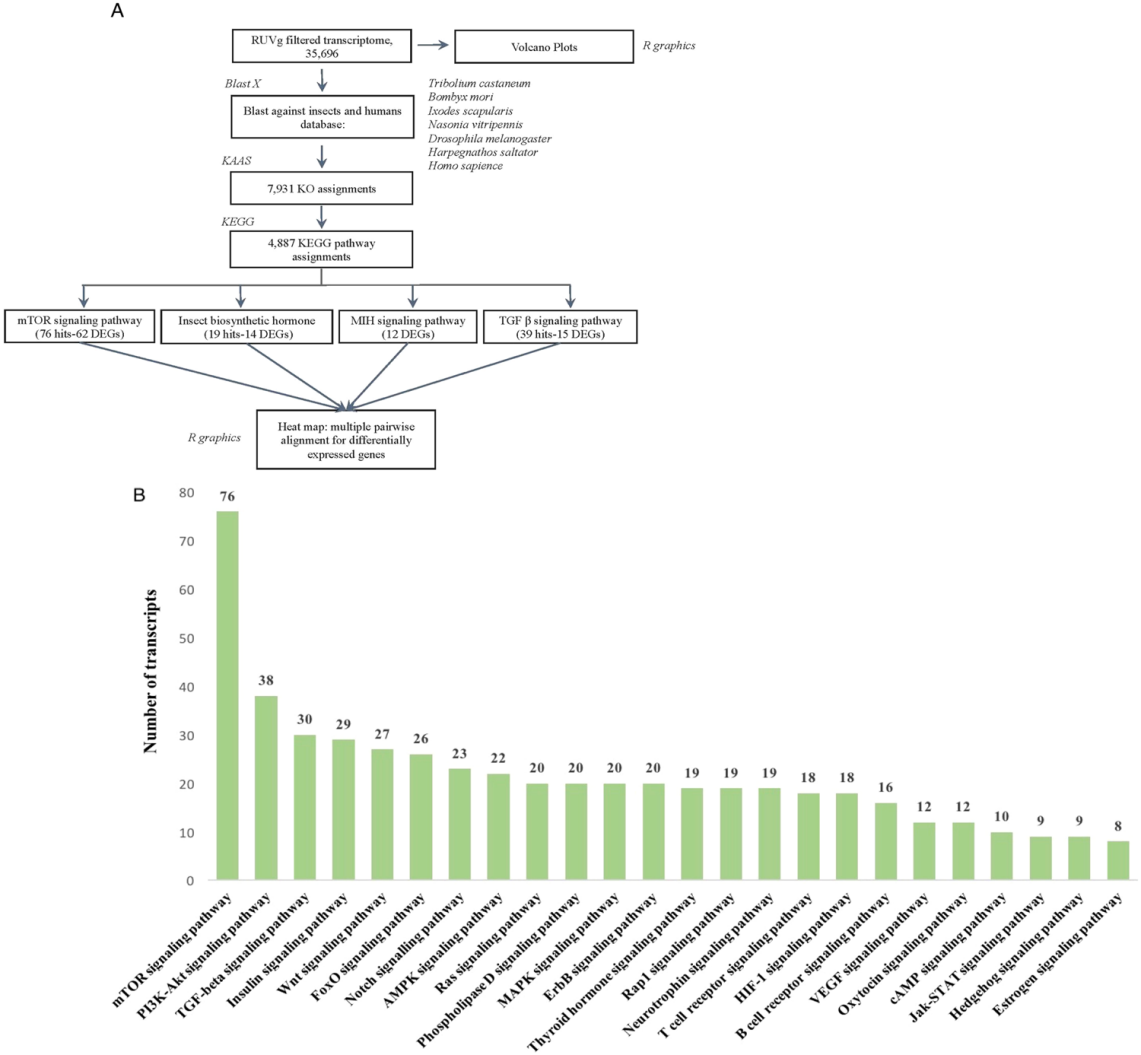
Functional characterization of YO transcriptome. (**A**) Pathway annotation pipeline using KAAS for KEGG pathway assignments. Six related insect species genomes (italics) were used for KO and pathway assignments. (**B**) Comparison of land crab YO contigs associated with selected second-tier KEGG pathways in the signal transduction category.

Pathway annotation for the filtered reference transcriptome (YO/I/DRESA_filtered; Fig. 2) was conducted via KASS (KEGG Automatic Annotation Server)^38^. KEGG orthology (KO) assignment and pathway annotation^39–41^ was performed on the data from Blastx analysis against 6 manually curated phylogenetically relevant insect species and the human (hsa) KO database (Section 4.5; Fig. 3A). The *G*. *lateralis* KO assignments were also compared to those from a YO transcriptome generated from the crayfish, *P*. *leptodactylus* (TSA: GAFS00000000)^42^. Following RUVg analysis, 35,696 YO/I/DRESA_filtered_transcriptome transcripts generated 7,931 hits to the insect databases; 2,340 had a KO assignment without functional annotation and 4,887 were annotated to one or more global KEGG pathway (Fig. 3A and Table 5). In addition, 464 were assigned to KO identifiers within 24 signal transduction pathways (Fig. 3B). As the KEGG pathways are manually curated, it is possible that unassigned KO hits are part of pathways that have not been identified in the database. The five highest pathway rankings based on KEGG assignment number were mTOR signaling, PI3K-Akt, TGFβ, insulin, and Wnt signaling pathways, with mTOR signaling having approximately a 2- to 2.5-fold higher number of assignments than in each of the other four (Fig. 3B).

#### Effects of rapamycin on YO ecdysteroidogenesis and gene expression

**Figure 4:**
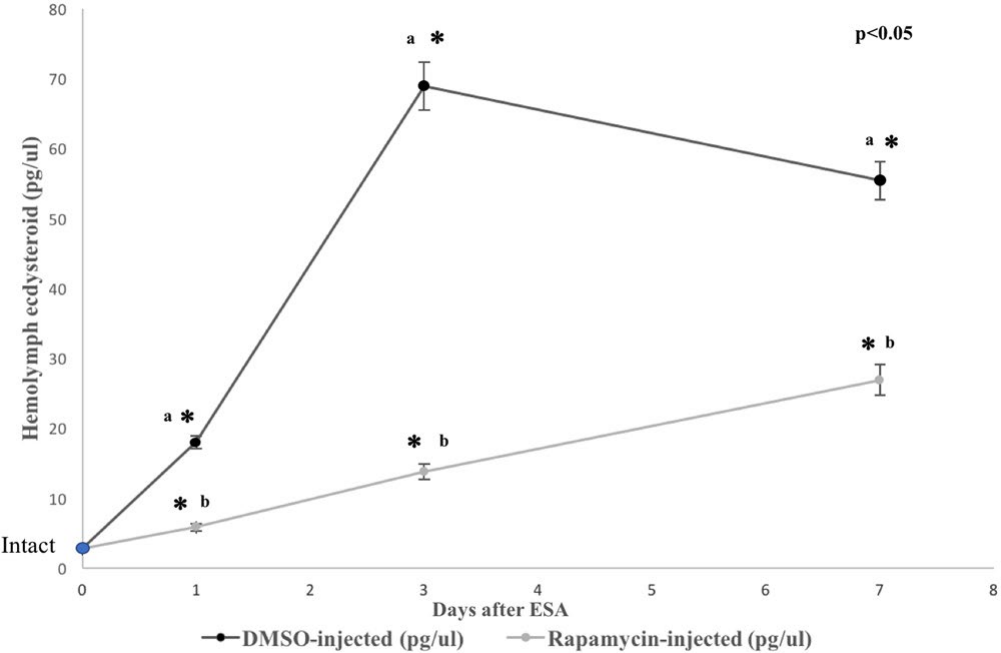
Hemolymph ecdysteroid titers prior and subsequent to ESA for DMSO- and rapamycin-injected animals. Intact (Intermolt) level represents the Day 0 reading (marked as the blue circle). The X axis represents days after ESA. Asterisks (*) represent significant differences between DMSO- and rapamycin-injected animals for each time point. Small letters represent significant differences within treatments between time points (a = DMSO-injected; b = rapamycin-injected).

ESA is a common method to induce entry into premolt in crustaceans via removal of circulating MIH^2^. Rapamycin treatment following molt induction blocks ecdysteroid synthesis in *G*. *lateralis*^13,14^ via inhibition of the mTOR protein kinase. Following ESA molt induction, although ecdysteroid levels increased for both DMSO- and rapamycin-treated animals, experimental animals treated with rapamycin showed a significant reduction in circulating hemolymph ecdysteroid, relative to DMSO-injected controls at 1, 3, and 7 days post-ESA (Fig. 4). There was a significant decrease in the control animals from 3 days to 7 days post-ESA. Although the YO was activated by ESA, the lack of increase in hemolymph ecdysteroid at day 7 indicates that the YO in control animals had not fully transitioned to the committed state by 7 days post-ESA. The significant decreases in hemolymph ecdysteroid titer as a consequence of rapamycin treatment indicated inhibition of YO entry into the activated state.

**Figure 5:**
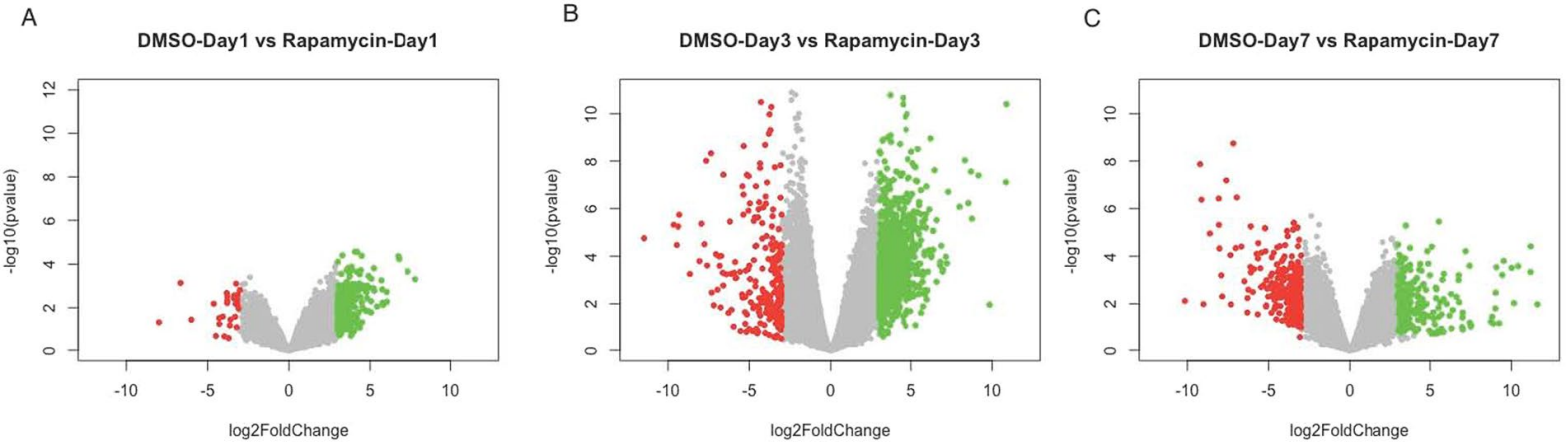
Volcano plots showing the pairwise comparison of differentially expressed genes (DEG) between DMSO-control and the rapamycin injected animals at Days 1 (**A**), 3 (**B**) and 7 (**C**). Up-regulated transcript abundance is indicated in green; down-regulated abundance in red. Highest numbers of DEGs were observed between DMSO-control vs rapamycin at Day 3.

Volcano plots of YO transcriptomes were developed from EdgeR pairwise comparisons of DMSO- *versus* rapamycin-injected animals at 1, 3 and 7 days following injection (Fig. 5). Rapamycin treatment, which has primarily been shown to affect regulation through post-transcriptional mechanisms, leads to both increases and decreases in transcript abundance relative to controls. Pairwise comparison showed that the greatest number of differentially expressed genes (DEGs) (3,104 genes up-regulated; 1,838 genes down-regulated) occurred at Day 3 (Fig. 5B). On Day 1 following rapamycin injection, a relatively smaller number of DEG contigs (764 genes up-regulated; 501genes down-regulated) showed differential expression (Fig. 5A). Day 7 comparisons showed a higher number of DEGs than Day 1, though less than at Day 3 (1,651genes up-regulated; 1,151 genes down-regulated) (Fig. 5C).

**Figure 6:**
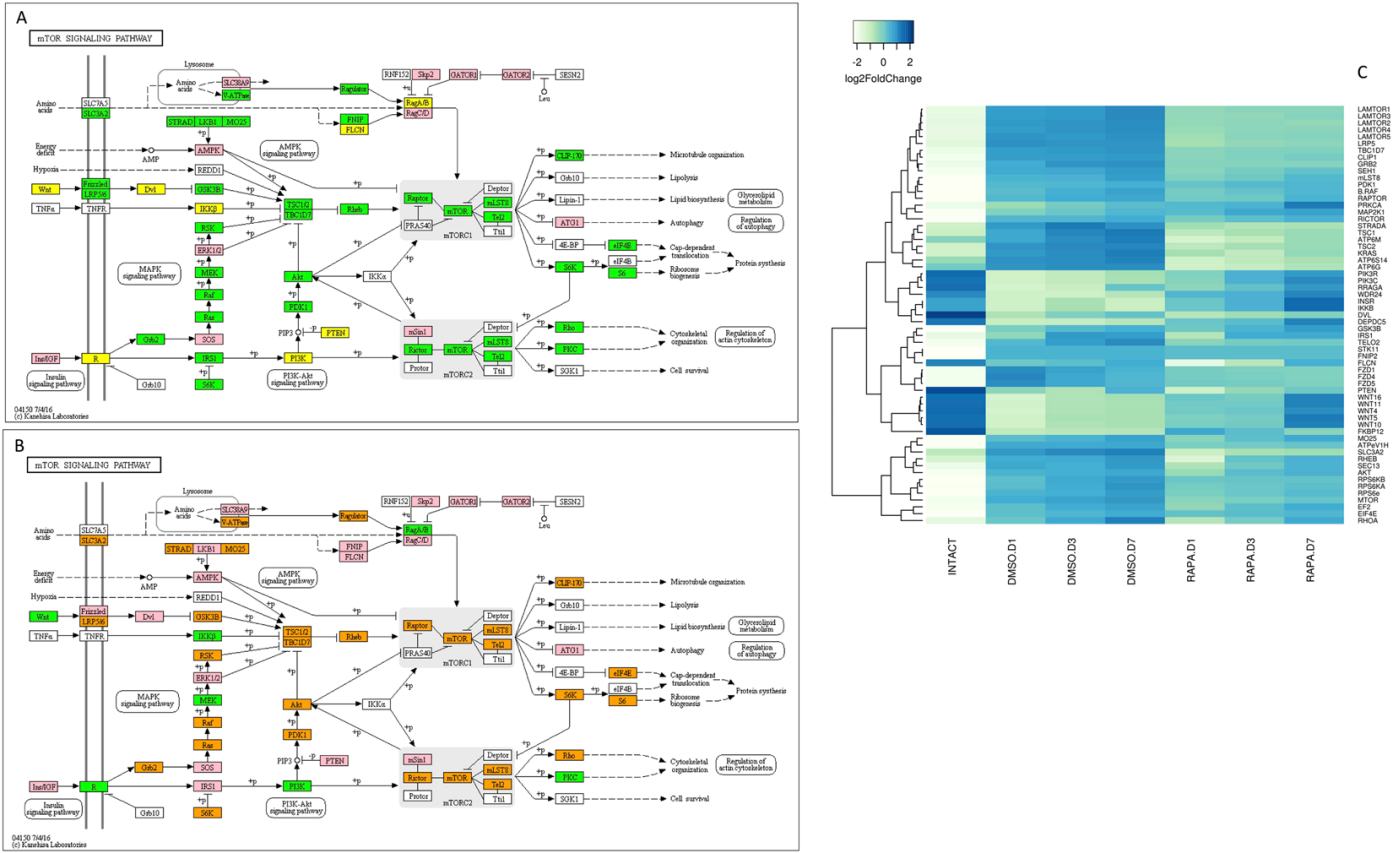
Identification of genes assigned to the mTOR/WNT/AMPK signaling pathway in the *G*. *lateralis* YO/I/DRESA transcriptome. In (**A**) and (**B**), KAAS was used to identify genes annotated by BLAST against the specific insect databases (section 4.5) and assigned to a KEGG pathway^38–40^. Uncolored boxes were genes not assigned during annotation. Pink colored boxes represent genes that show no change in expression levels between pairwise comparisons within the 5A and 5B datasets. (**A**) Pairwise comparison of intact/intermolt vs ESA DMSO-control animals. Green colored boxes represent genes up-regulated in premolt (D libraries) relative to intermolt (intact); a yellow color indicates genes down-regulated when entering premolt relative to intermolt. (**B**) Pairwise comparison of ESA DMSO- and Rapamycin-injected animals. Orange colored boxes indicate genes in premolt (D libraries) that decrease in transcript abundance consequent to rapamycin treatment (R libraries); green represents genes normally down-regulated when entering premolt that are now up-regulated by rapamycin treatment. (**C**) Heat map showing the log2-fold change of the FPKM values of the DEGs.

Pathway analysis compared changes in gene transcript abundance between (1) intact (intermolt) to ESA control (premolt) animals and (2) premolt DMSO control to rapamycin-treated (mTOR-inhibited) animals. qRT-PCR experiments have shown that during ESA-induced molt in *G*. *lateralis*, mRNA levels of *Gl*-*mTOR*, *Gl*-*Akt*, and *Gl*-*S6K* are increased relative to intermolt^14^. This is in accordance with the current transcriptomic analyses (Fig. 6 and Suppl. Figure 5A,B). DEG analysis revealed increased mRNA levels for a large number of mTOR pathway genes following ESA (Fig. 6C and Suppl. Figure 5A,B). When intact (intermolt) animals were compared to premolt DMSO control animals, increases in transcript abundance were observed for all of the major mTOR signaling pathway genes, such as *mTOR*, *mLST8*, *Tel2*, *Raptor*, *Rictor*, *Akt*, *Rheb*, *Ras1*/*2*, *Raf*, *MO25*, *RhoA*, *PDK1*, *Sec13*, *EIF4E*, and *EF2* (Fig. 6A,C and Suppl. Figure 5A,B); this was also correlated with increases in ecdysteroid titer (Fig. 4). By contrast, rapamycin significantly decreased transcript abundance of the mTOR pathway genes (Fig. 6B,C and Suppl. Figure 5A,B). In rapamycin-treated animals, mRNA levels for *mTOR*, *mLST8*, *Tel2*, *Raptor*, *Rictor*, *Akt*, *Rheb*, *Ras1*/*2*, *Raf*, *MO25*, *Rho1*, *PDK1*, *Sec13*, *ElF4E*, and *EF2* decreased at Day 1 and then began to recover by Day 7, although the mRNA levels remained below those in the DMSO controls (Fig. 6C and Suppl. Figure 5B).

ESA induced entry into premolt also led to an increase in transcript abundance for several other genes involved in mTOR signaling pathway regulation, as well as genes involved in down-stream effects on cell division and protein synthesis^19,22^. In Fig. 6C, a group of genes in the DMSO control animals were up-regulated as a result of ESA in comparison to the intact animals in intermolt: *CLIP1*, *TBC1D7*, *TSC1*/*2*, *Grb2*, *STRADA*, *LRP5*/*6*, *SLC3A2*, and the ragulator complex, which is composed of *Lamtor1*/*2*/*3*/*4*/*5*. Rapamycin lowered mRNA levels for these genes with respect to control animals (Fig. 6B,C and Suppl. Figure 5C,D).

In transitioning from intermolt (intact animals) to premolt (DMSO animals), several genes, such as *Wnt*, *Dvl*, *INSR*, *IKK*β, *PTEN*, *FLCN*, and *FKBP12*, were strongly down-regulated by ESA (Fig. 6A and Suppl. Figure 5A,B,E and F). FKBP12 binds rapamycin and inhibits mTOR protein kinase activity^19,22^. The highest level of mRNA expression was observed for *FKBP12* in intact, intermolt animals as compared to premolt DMSO control animals. Entry into premolt led to a greater than 300-fold decrease in *FKBP12* transcript abundance in DMSO controls, and mRNA levels increased approximately 10-fold following rapamycin treatment, relative to DMSO control levels. For most genes subject to rapamycin inhibition, transcript levels tended to increase over time (Fig. 6C and Suppl. Figure 5A,B). However, for several pathway genes upstream of mTOR signaling, such as *Wnt*, *INSR*, and *IKKβ*, we observed a significant increase in mRNA levels in premolt rapamycin-treated animals relative to DMSO controls, which persisted and increased through to Day 7 post-ESA. In addition, several genes that decreased in transcript abundance when entering premolt, were increased by rapamycin treatment (e.g., *PIK3C*, *PRKCA*, *RRAGA*, *WDR24*, and *DEPDC5*) (Fig. 6C and Suppl. Figure 5E,F).

#### Insect hormone biosynthetic pathway (IHB)

**Figure 7:**
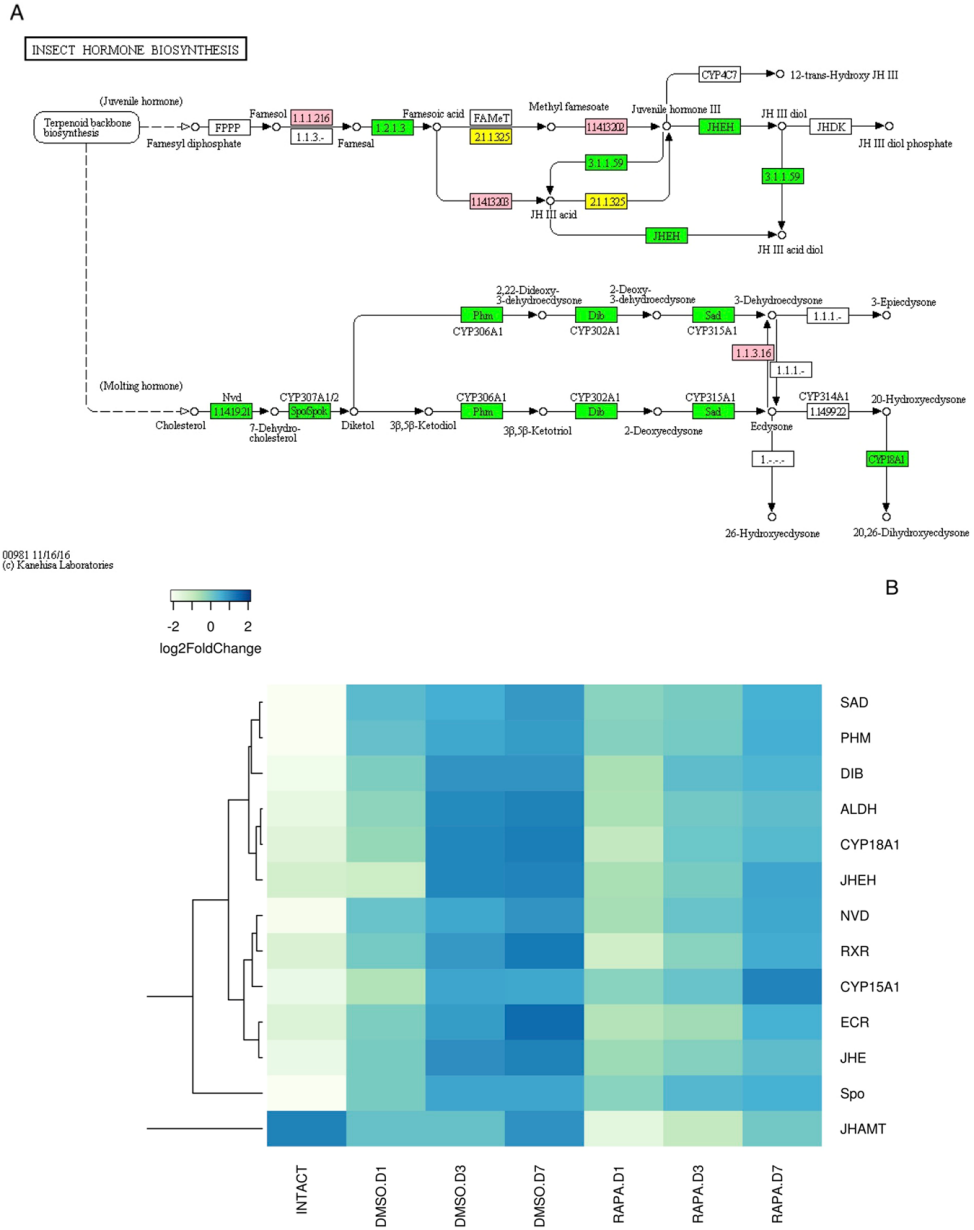
Identification of genes assigned to the insect hormone biosynthetic (IHB) pathway in the *G*. *lateralis* YO/I/DRESA transcriptome. KAAS was used to identify genes annotated by BLAST against the specific insect database (Section 4.5) and assigned to a KEGG pathway^38–40^. (**A**) IHB pathway components/genes. Colored boxes indicate the presence of the genes identified in the ESA database. A pink colored box signifies the presence of an annotated gene that does not show a change in expression levels; a green box indicates a significant increase in premolt transcript abundance relative to intermolt while a yellow box indicates a decrease in transcript abundance. (**B**) Heat map showing the log2-fold change of the FPKM values of the DEGs.

The effects of ESA and mTOR pathway inhibition on the expression of insect IHB genes were also examined. IHB genes were assigned to the ecdysteroid and juvenile hormone (JH) biosynthetic pathways using KASS analysis (Fig. 7). In intact (intermolt) animals, low mRNA expression levels were observed for the ecdysteroidogenic genes *Phm*, *Sad*, *Dib*, *Nvd*, and *Spo*, as well as the ecdysteroid receptor heterodimer (*EcR*/*RXR*). ESA increased transcript levels for these genes, with the highest mRNA levels in Day 7 control DMSO (premolt) animals (Fig. 7 and Suppl. Figure 5G). Although the relative expression of all the ecdysteroidogenic genes increased significantly from 1 day to 7 days post-ESA, the absolute expression of the *spo* gene (Cyp307a1), a gene involved in the stage 1 black box reactions leading to dikediol^43^, exhibited transcript levels approximately 25- to 35-fold higher than the stage 1 *nvd* gene or the stage 2 Halloween gene hydoxylases (*phm*, *dib* and *sad*) in the pathway (Suppl. Figure 5G). Rapamycin injection reduced the mRNA levels of the ecdysteroidogenic and JH pathway genes (Fig. 7B and Suppl. Figure 5G). *Phm*, *sad*, *dib*, *nvd*, *spo*, and *EcR* mRNA levels recovered by day 7 (Fig. 7B and Suppl. Figure 5G), again suggesting a diminishing inhibitory effect on the mTOR pathway from the single rapamycin injection.

Contigs encoding ten genes in the JH synthetic pathway were identified in the YO transcriptome. ESA resulted in significant changes in seven of the 10 genes; five showed increased mRNA levels, while two showed decreased mRNA levels (Fig. 7A). One of the down-regulated contigs encoded a gene at the end of the sesquiterpenoid pathway, juvenile hormone acid O-methyltransferase (*JHAMT*), which catalyzes the conversion of farnesoic acid to its cognate methyl ester, a proposed crustacean JH analog^44^. Transcript levels for this gene were highest during intermolt; there was approximately a 4.5-fold decrease in transcript levels for *JHAMT* in premolt DMSO animals relative to intact intermolt. Transcript levels increased during premolt, reaching 70% of intermolt levels by 7 days post-ESA (Fig. 7B and Suppl. Figure 5G).

#### Molt-inhibiting hormone (MIH) signal transduction pathway

**Figure 8:**
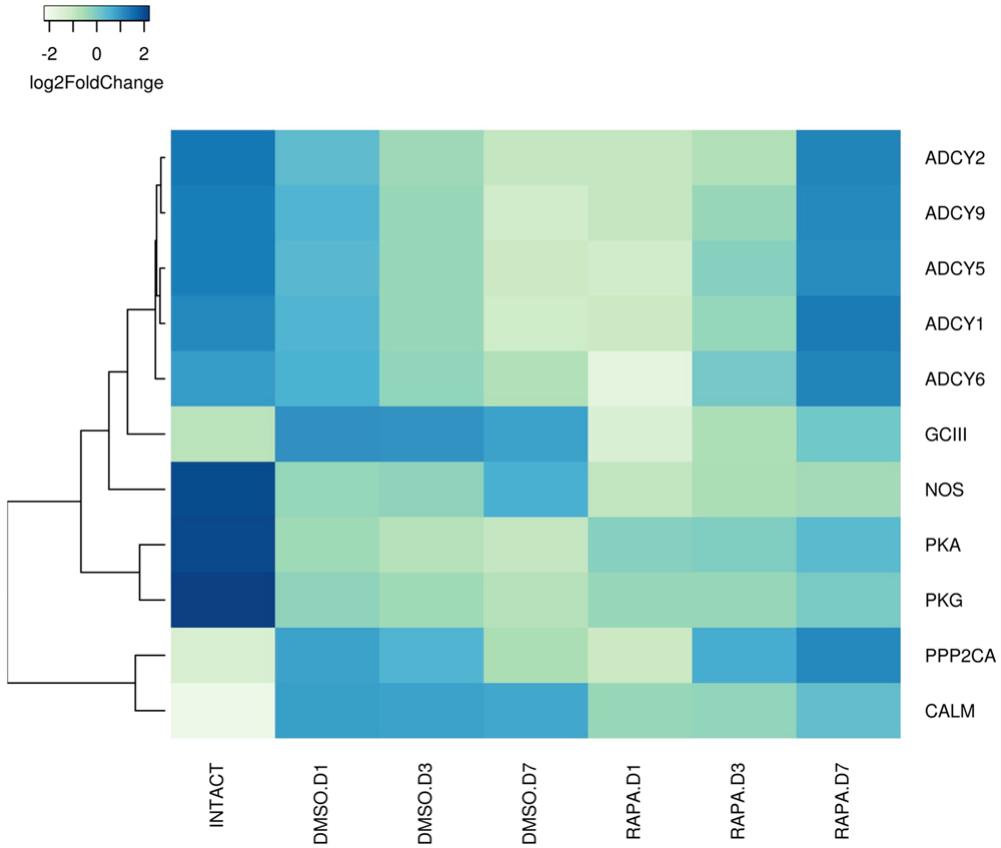
DEGs in the *G*. *lateralis* YO/I/DRESA transcriptome implicated in MIH signaling pathway^5,7,11^. Heat map showing the log2-fold change of the FPKM values of the DEGs. KAAS was used to identify genes annotated by BLAST against the specific insect database (Section 4.5) and assigned to a KEGG pathway^38–40^.

KO identifiers for putative molt inhibiting hormone (MIH) pathway genes were extracted from the KEGG database. The genes catalogued as MIH pathway genes were identified through literature survey^5,29,45^. KEGG analysis generated annotated YO gene assignments and DEG determinations from mRNA transcript abundance measurements were identified via ANOVA. The highest mRNA transcript levels were observed in the intermolt/intact animals for the genes encoding adenylyl cyclase (*ADCY1*, *ADCY2*, *ADCY5*, *ADCY6*, and *ADCY9*), nitric oxide synthase (*NOS*), protein kinase G (*PKG*), and protein kinase A (*PKA*) (Fig. 8 and Suppl. Figure 5H). As the control (DMSO-injected) animals entered premolt, a progressive decrease in transcript levels were observed for these genes (Fig. 8 and Suppl. Figure 5H). A different expression pattern was observed for NO-insensitive soluble guanylyl cyclase (*GC-III*), calmodulin (*CaM*), and serine/threonine-protein phosphatase 2A (*PPP2CA*). These genes showed initial increases in transcript abundance in transitioning from intermolt to premolt (Fig. 8 and Suppl. Figure 5H). Also identified were other MIH pathway genes^29^^,^^44^; calcineurin (*CaN*), NOS interacting protein (*NOSIP*), NO-sensitive guanylyl cyclase (*GC-Iβ*), and protein kinase C (*PKC*). Transcript levels for these genes were indistinguishable from intermolt levels through day 7 following ESA (data not shown).

The effects of rapamycin varied between the MIH signaling genes. For *GC-III* and *CaM*, the effect was similar to that observed for many of the genes in other pathways: relative to controls, transcript levels decreased immediately following drug injection and increased by Day 7 (Fig. 8 and Suppl. Figure 5H). For the five *ADCYs*, *PP2CA*, and *PKA*, however, transcript levels were marginally reduced or unaffected relative to controls at Day 1, but increased significantly by Day 7 in rapamycin-treated animals (Fig. 8 and Suppl. Figure 5H). For *ADCYs* and *PP2CA*, these approached or exceeded the mRNA levels observed in intermolt animals. For *NOS*, rapamycin injection led to a small significant decrease in transcript levels only by Day 7 (Fig. 8 and Suppl. Figure 5H). Rapamycin had no significant effect on the mRNA levels of *CaN*, *NOSIP*, *GC-Iβ*, and *PKC* (data not shown).

#### TGFβ signaling pathway

**Figure 9:**
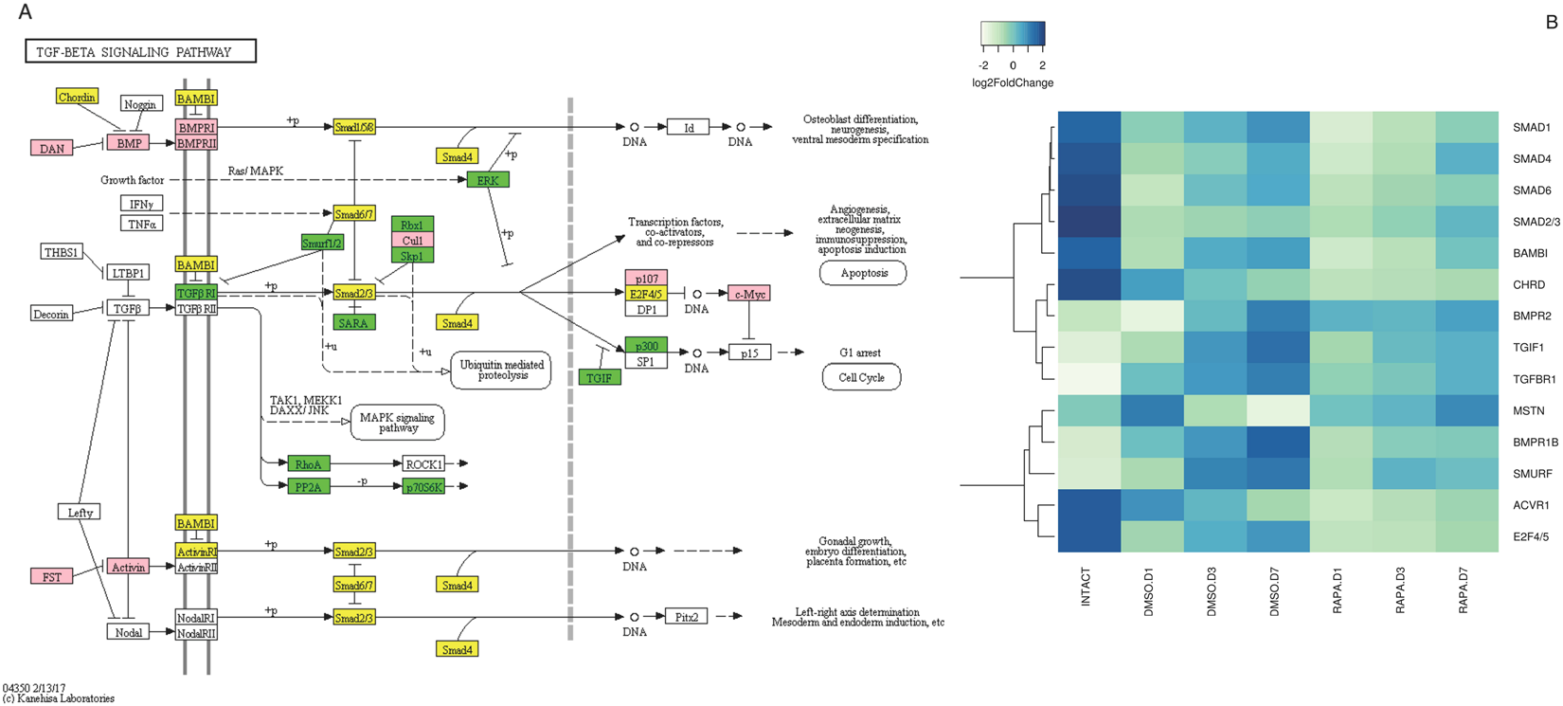
Identification of genes assigned to the TGFβ/Activin signaling pathway in the *G*. *lateralis* YO/I/DRESA transcriptomes. KAAS was used to identify genes annotated by BLAST against the specific insect database (Section 4.5) and assigned to a KEGG pathway^38–40^. (**A**) TGFβ signaling pathway components/genes. Colored boxes indicate the presence of the genes identified in the ESA database. A pink colored box signifies the presence of an annotated gene that does not show a change in expression levels between intermolt and premolt; a green box indicates a significant increase in premolt transcript abundance relative to intermolt while yellow box indicates a decrease in transcript abundance. (**B**) Heat map showing the log2fold change of the FPKM values of the DEGs.

TGFβ signaling genes were identified in the *G*. *lateralis* transcriptome through homology to insect databases; both the BMP and Activin pathway genes were expressed (Fig. 9A). Transcript abundance for contigs encoding TGFβ family receptors *TGFßR1* and *BMPR1*, *SMURF E3 ubiquitin ligases*, and transcription factor *TG1F* increased relative to intermolt levels through Day 7 in control animals, while the mRNA level of a contig encoding *BMPR2* was unchanged (Fig. 9B and Suppl. Figure 5I). Rapamycin lowered the mRNA levels of *BMPR1*, *TGFβR1*, *TG1F*, and *SMURF* relative to the DMSO control, while having no effect on the *BMPR2* mRNA level (Fig. 9B and Suppl. Figure 5I). Transcript levels of *Chordin*, an inhibitor of BMP, and *BAMBI*, an inhibitor of TGFβ receptors (Fig. 9A), were significantly higher in intermolt animals. Transcript levels of contigs encoding receptor-regulated Smad (*Smad1*/*2*/*3*), common-mediator Smad (*Smad4*), and inhibitory Smad (Smad6/7) also decreased approximately 25–50% from intermolt to entering premolt. The mRNA levels for *BAMBI* and *Smads 1*, *4* and *6* generally exhibited an increase in transcript abundance proceeding from day 1 through day 7 of premolt. While rapamycin treatment significantly lowered transcript levels for these genes, this pattern of transcript abundance increasing as premolt proceeded was maintained (Fig. 9A and Suppl. Figure 5I).

In contrast to some components of the TGFβ/BMP pathways showing up-regulation entering premolt or recovery as premolt proceeded, several genes in the Activin pathway were either unchanged or down-regulated in control animals (Fig. 9A). The mRNA level of a contig encoding Activin receptor *ACVR1* decreased during premolt and there were no significant changes in the Activin-like *MSTN* transcript level. Although all *Smad* genes showed a significant decrease in transcript levels on entering premolt, receptor-regulated *Smad2*/*3*, *E2F4*/*5* and the Activin-like *MSTN* transcript levels were unaffected by rapamycin treatment (Fig. 9B and Suppl. Figure 5I).

## Discussion

Previous studies have implicated the mTOR pathway in mediating YO transcriptional responses associated with YO activation during premolt^13,14^ and we examined in detail mTOR signaling genes within the KO reference pathway. We also focused on other pathways that interact with mTOR signaling, specifically the Wnt, AMPK, insulin signaling, and insect biosynthetic hormone pathways. Finally, we examined second messenger pathways that had previously been implicated in mediating MIH inhibition of ecdysteroid biosynthesis and the transition from intermolt to the activated premolt state, as well as the TGFβ signaling pathway that mediates the transition of the YO from the activated to committed states at mid-premolt^5,12–14^. RNA-seq was used to quantify gene expression in the YO as it transitioned from the basal state in intermolt to the activated state in early premolt^5^. The changes in hemolymph ecdysteroid titer indicated that the YOs were activated by ESA in the controls and that rapamycin inhibited YO activation, as reported previously^14^. However, unlike the previous study, where hemolymph ecdysteroid titer continued to increase at Day 7 post-ESA^14^, there was a significant decrease in titer from Day 3 to Day 7 (Fig. 3), indicating that the YOs were not generating the high hormone levels associated with the committed state.

mTOR is a component of the phosphatidylinositol 3-kinase (PI3K) cell survival pathway that monitors nutrient availability, mitogen-activated signal transduction, and cellular energy and oxygen levels; it is critical in the regulation of cell growth and proliferation in all metazoans^19,22^. mTOR can form two multiprotein complexes, mTORC1 and mTORC2, that regulate protein synthesis necessary for cell growth and proliferation. mTORC1 is inhibited by the antibiotic rapamycin through forming a complex with immunophilin FKBP12^46–48^. Although the mode of action is unclear, inhibition may occur through blocking phosphorylation of selective substrates. Active mTORC1 exerts numerous downstream biological effects, including the translation of mRNA by phosphorylating downstream targets, such as 4E-BP1 and S6K, and the suppression of autophagy through Atg13 and ULK1^22,49,50^. In some cell types, prolonged exposure to rapamycin variably affects the formation of new mTORC2 complexes, but apparently not existing complexes^49^.

The mTOR catalytic domain has a two-lobed structure with catalytic loop and activation loop motifs characteristic of the PIKK family^51,52^. Phosphorylation of a serine and a threonine, located near the amino terminus of the kinase domain, is associated with mTOR activation^53^. This site is highly conserved between *G*. *lateralis* mTOR and insect mTOR^13^. There is conclusive evidence of the ability of rapamycin to interact with mTOR homologs in arthropod systems. Rapamycin inhibits protein synthesis in *Drosophila* S2 cells^22,54^. In *Bombyx mori*, rapamycin inhibits PTTH-dependent phosphorylation of S6K and 4E-BP1 and ecdysteroid synthesis and secretion^55^. In *Manduca sexta*, feeding rapamycin to larvae delays molting and reduces growth and ecdysteroid production by the PG^56^. In *G*. *lateralis*, rapamycin inhibits YO ecdysteroid secretion *in vitro* and hemolymph ecdysteroid titers *in vivo*^13,14^. These data indicate that activation of the molting gland requires mTORC1 activity in both insects and crustaceans. In insects, the regulation of body size, antimicrobial defense, responses to starvation-induced dormancy and aging are mediated by mTOR signaling^57–61^.

Genes encoding *mTOR*, *Rheb*, *Akt*, and *S6K* orthologs were previously characterized in *G*. *lateralis*^13,29,62^ and additional pathway components were identified in this study (Fig. 6A). The deduced sequences showed high sequence identity and domain organization to orthologs from insects and other species^13,62^. *mTOR*, *Rheb*, *Akt*, and *S6K* are expressed in every tissue monitored, indicating that this pathway has essential cellular functions^13,63^. The mTOR pathway is transcriptionally up-regulated by ESA in control animals, as there were large increases in the mRNA levels for a large number of mTOR pathway components (Fig. 6 and Suppl. Figure 4A,B). This supports earlier studies^13,14^ implicating mTOR pathway function in the activated YO. mTOR genes that showed the greatest increase in transcript abundance were *mTOR1*/*2*, *EF2*, *EIF4E*, *RhoA*, *TSC1*/*2*, *S6K*, *S6*, *Mo25*, *Akt*, and *Rheb* (Fig. 6 and Suppl. Figure 1B,D). Akt is the central mediator of protein synthesis and cell survival in response to growth factors^63^ and Rheb is a crucial upstream activator of mTOR^64,65^.

As noted above, previous studies have shown that mTOR activity is required for increased ecdysteroidogenesis in activated YOs released from MIH inhibition^13,14^. This suggests that MIH inhibits mTORC1 activity. Although the regulation of protein synthesis by mTORC1 relies on post-translational protein interactions mediated through phosphorylation and GTP hydrolysis, this study demonstrates that MIH signal transduction also affects transcriptional expression of numerous genes involved in both ecdysteroid biosynthesis, as well as components of the mTOR signal transduction pathway. In parallel with increases in YO ecdysteroid secretion, DEG analysis showed increases in transcript levels for genes involved in ecdysteroid synthesis from cholesterol (*phm*, *sad*, *dib*, *nvd*, and *spo*), as well as for the ecdysteroid receptor (*EcR*, *RXR*) (Fig. 7 and Suppl. Figure 5G). Relative to controls, rapamycin injection most often led to decreases in mRNA levels for genes in both the mTOR and ecdysteroid synthesis pathways, which often began to recover over the 7-day monitoring period (Fig. 6 and Suppl. Figure 4B). Since only a single antibiotic injection was administered, this recovery may reflect drug stability and half-life. Although the pharmacokinetics of rapamycin has not been explored in invertebrate systems, drug clearance shows non-linear kinetics and has a half-life of approximately 60 hours in human adults^66,67^. Taken together, the results show that mTORC1 activity increases the ecdysteroidogenic capacity of the YO by up-regulating ecdysteroid biosynthetic pathway genes.

Interestingly, transcript levels of the *spook* gene, a class 1 monooxygenase involved in diketol synthesis from cholesterol, increased 25- to 35-fold relative to the other ecdysteroid pathway enzymes following entry into early premolt. qPCR studies in the swimming crab, *Portunus trituberculatus*, did not report an increase in *spo* transcripts on entering premolt^68^. In insects, however, a homolog of this gene, *spookier*, is subject to transcriptional up-regulation by the zinc finger transcription factor Ouija board (*ouib*), which is principally expressed in the ecdysteroidogenic prothoracic gland^69,70^. Several zinc-finger proteins are expressed in the *G*. *lateralis* YO, but it is currently unclear if any are predominantly expressed in this tissue (Shyamal *et al*., unpublished). Whether this enzyme may be rate-limiting for ecdysteroid synthesis, as proposed in insects, and what additional factors contribute to transcriptional regulation of ecdysteroidogenesis during YO commitment requires further study^71,72^.

Not all transcripts, however, followed a pattern of a decrease in mRNA levels following rapamycin administration, suggesting that mTOR signaling represses gene expression. The gene encoding the protein responsible for binding rapamycin, *FKBP12*, showed increased transcript levels following rapamycin treatment, starting high then declining over time (Fig. 6 and Suppl. Figure 5B). This may reflect a transcriptional up-regulation due to rapamycin treatment that declines as drug potency wanes. Interestingly, several genes showing increases in transcript abundance showed a reciprocal pattern of transcript accumulation from that observed for *FKPB12*. Notably, genes encoding Wnt growth factors, the insulin receptor, and PI3K showed progressively increasing mRNA levels in rapamycin treated animals, remaining highly elevated relative to ESA premolt controls, even at Day 7 (Fig. 6C and Suppl. Figure 5F). Wnt (Fig. 6), an activator of mTORC1, has been implicated in ecdysteroidogenesis in the insect prothoracic gland^73^. These genes may undergo increased initial rates of transcription due to rapamycin treatment. The continued increase may be due to positive transcriptional feedback up-regulation by these growth factors. Transcripts encoding transcription factors associated with both the canonical Wnt pathway (TCF/LEF/pangolin/HMG box superfamily; groucho/WD40 superfamily/) and insulin-like signaling pathway (FOXO/forkhead related) have been identified in the YO Illumina libraries under differing physiological conditions, such as limb regeneration, over the molt cycle (Shyamal *et al*., in preparation). The ability to identify and quantify these components, and the potential to manipulate pathway signaling, will aid in the examination of their role in mediating YO physiological responses during growth and regeneration.

Unlike the positive PTTH effector identified in insect systems, the inhibitory neuropeptide MIH suppresses ecdysteroidogenesis in crustaceans. Although the regulatory pathway mediating MIH signaling has not been clearly elucidated, previous work hypothesizes that a membrane receptor triggers downstream cyclic nucleotide effectors leading to inhibition of hormone biosynthesis^4,5,11,12^. Searching our database for sequence similarity to known cyclic nucleotide pathway components, we identified orthologous pathway candidates^45^. Most of the putative pathway components decreased in transcript abundance on entry into premolt (Fig. 7), which correlates with a reduced sensitivity to MIH by the activated YO^5,11^. As in the other pathways analyzed, however, both increases and decreases in gene transcription could be attributed to rapamycin treatment, dependent on the gene. As noted above, identification of potential pathway components at the nucleotide sequence level now affords the ability to examine their possible roles in the molecular mechanism of MIH action. Evidence is accumulating that mTORC1 regulates expression at the transcriptional level, as well as at the translational level^74^. The data suggest that mTORC1 activity, either directly or indirectly, controls transcription of genes involved in YO activation.

In summary, transcriptomics revealed large changes in gene expression and established a central role for mTOR in YO activation. Rapamycin altered the mRNA levels of thousands of genes (Fig. 5). Both the mTOR and TGFβ signaling pathways have been shown to play important roles in the control of ecdysteroidogenesis in insect systems^24,25,27,55,56^. Previous work in *G*. *lateralis* has shown that inhibition of mTOR (using rapamycin) or TGFβ (using SB431542) signaling decreases YO ecdysteroidogenesis^13,14^. Neuropeptides, such as PTTH in insects and MIH in decapod crustaceans, control ecdysteroidogenesis in their respective molting glands either by stimulating mTOR activity in the insect prothoracic gland or by inhibiting mTOR activity in the crustacean YO^12,55,56^. Significant decreases in mRNA levels of key components of the proposed MIH signaling pathway, such as five *ADCY*s, *PKA*, *NOS*, and *PKG*, by 3 days post-ESA (Fig. 7), may contribute to the reduced sensitivity of the YO to MIH in mid and late premolt^5,11^. Interestingly, rapamycin did not block or reverse the decrease in MIH signaling genes from their intermolt transcript levels until Day 7 for most genes (*ADCY1*, *2*, *5*, *6*, & *9*, *PKA*, and *PKG*; see Fig. 8), suggesting an ESA-induced hysteresis in the YO sustained the down-regulation of these genes during activation during premolt, and, with potential loss of rapamycin potency, transcript levels increased. We demonstrate here that ESA up-regulates many mTOR signaling genes, which include the MAPK and PI3K pathways (Fig. 6), and genes involved in ecdysteroid synthesis (Fig. 7), which together would contribute to the increased ecdysteroidogenic capacity of the YO in premolt animals^5^. Rapamycin inhibits the ESA-induced increases of the mTOR signaling genes (Fig. 6), which suggests a positive feedback loop in which mTOR drives YO activation. The ecdysteroid biosynthetic pathway appears to be a primary target of mTOR, as rapamycin counters the increased expression of ecdysteroidogenic genes (Fig. 7). Interestingly, ESA did not up-regulate expression of genes in the Activin/TGFβ pathway. In a previous study, ESA increased *Gl-Mstn* mRNA level in the YO 5.5-fold by 3 days^14^. In the current study, *Gl-Mstn* transcript levels were unaffected by ESA and ESA down-regulated other genes in the pathway (Fig. 9; e.g., *ACVR*1, *Smad 1*, *Smad 2*/*3*, *Smad* 4, and *Smad* 6). It may be noteworthy, however, that transcript levels of both TGFß pathway inhibitors (e.g. *BAMBI*, *Smad* 6), and pathway effectors (e.g. *Smad 1*/*4*) began to recover as premolt proceeded from day 1 through day 7. Since ecdysteroid levels at day 7 of premolt did not show a sustained increase in titer, the difference between the studies may be linked to animals in this study not transitioning to the committed state by 7 days post-ESA (Fig. 4). The transcriptional consequences of TGFβ pathway inhibition by SB431542 on the YO remain to be investigated in animals transitioning to the committed state.

## Methods

### Animals

Adult male *G*. *lateralis* were collected in the Dominican Republic, shipped to Colorado State University, and maintained as described^14^. Molting was induced by eyestalk ablation (ESA)^2^. Rapamycin (an inhibitor of mTOR; Selleck Chemicals, Houston, TX, USA), as well as control DMSO carrier injections were conducted immediately following eyestalk ablation as previously described^14^. Twenty-seven animals were designated controls and injected with 0.3 µl/g body weight of DMSO carrier (DA, DC, DG). Another set of 27 animals was injected with a similar volume of DMSO carrier at a rapamycin concentration of 10 mM (RA, RC, RG). Y organs (YOs) were collected on days 1, 3 and 7 for both injection control and experimental sets. Nine control intermolt animals were not subjected to ESA and did not undergo an injection protocol. YOs were dissected from the branchial chamber side of the anterior branchiostegite region of the cephalothorax^1^, then stored in 300 μl RNAlater (Life Technologies, Grand Island, NY, USA) at −80 °C until processing. Hemolymph ecdysteroid titers at the time of dissection were quantified using a competitive enzyme-linked immunosorbent assay (ELISA)^75,76^. YOs were transported to the University of Oklahoma in 4 °C storage boxes and then stored at −20 °C until mRNA isolation.

### mRNA isolation, library preparation, and sequencing

Total RNA was isolated using RNeasy™ Mini Kits (Qiagen, Valencia, CA, USA) following the manufacturer’s protocol. RNA was quantified using Qubit Fluorometric Quantitation (Thermo Fisher Scientific, USA). Thermo-Fisher ERCC ExFold RNA Spike-in Control Mix 1 and Mix 2, which mimic 92 natural eukaryotic mRNAs and aid in transcript quantification normalization, were added according to the manufacturer’s protocol. mRNA purification and cDNA synthesis was carried out with a TruSeq™ Stranded mRNA Library Prep Kit (Illumina). Twenty-one cDNA libraries were generated; with the following naming convention: IN: Intact animal YO (intermolt); DMSO injected; DA = DMSO injected YO on day 1, DC = DMSO injected YO on day 3, DG = DMSO injected YO on day 7; Rapamycin injected: RA = Rapamycin injected YO on day 1, RC = Rapamycin injected YO on day 3, RG = Rapamycin injected YO on day 7. Three biological replicates for each experiment were performed (e.g. represented as DA1–3, respectively). Each library was derived from mRNA from six YOs pooled from three animals. Paired-end sequencing of the cDNA libraries using an Illumina HiSeq™ 3000 instrument was performed at the Oklahoma Medical Research Foundation. All samples were run in 7 sequencing lanes with 21 adaptor tags.

### Quality control and transcriptome assembly

The quality of paired-end raw reads in Fastq format was assessed using the FASTQC program (Babraham Institute, Cambridge, UK). Reads with a minimum Phred (nucleotide base call) quality score of 28 and length ranging from 36 bp to 100 bp were extracted by trimming of low quality reads and adaptor sequences via Trimmomatic software (version number: 0.32)^77^. The trimmed reads obtained from the ESA replicates were concatenated into two files containing forward (ESA_paired-FR.fa) and reverse (ESA_paired-RV.fa) sequences, separately. A BioSample data set (SRP132101) has been submitted to the NCBI sequence read archive: https://trace.ncbi.nlm.nih.gov/Traces/sra/sra.cgi?view=announcement.

The trimmed forward and reverse reads were then assembled via Trinity software with default settings (version number: 2.0.1)^30^. The minimum contig length was set at 201 bp. Following assembly, the contigs were clustered based on a 95% sequence similarity threshold using the CD-HIT-EST program (version number: 4.6.1)^31^ (Fig. 2). The assembled transcriptome is available in fasta format in the Cyverse Discovery environment at: https://de.cyverse.org/dl/d/915A4462-D13E-443C-88FE-30C1C7686E50/ESA_reference.fasta.

### Transcript abundance, filtering and RUVg normalization

Since the fully annotated genome of a crab species has not been published, the transcript abundance for each library was accomplished in two steps. First, the CD-HIT assembled transcriptome was mapped back to the concatenated forward (ESA_paired-FR.fa) and reverse (ESA_paired-RV.fa) raw reads using Bowtie2 (version number: 2.2.9)^32^ to generate a SAM (Sequence Alignment/Map) file. SAMtools software (version number: 1.3) was used to convert the SAM files to BAM (binary form of SAM file) files. The BAM files were used to quantify mapped transcript abundance for individual libraries using eXpress software (version number: 1.5.1)^78^. Low expression contigs were filtered out using edgeR filtering^33^. Expression levels are defined as counts per million (CPM). Contigs with low expression were filtered out using CPM of 3 in all the three replicates. Here, a CPM of 1 corresponds to a count of 6–7 in the smallest sample. Contigs are dropped if they were not expressed in any of the 2 replicates for any of the conditions.

The filtered database was used as the reference transcriptome and was designated as YO/I/DRESA (Fig. 2). The YO/I/DRESA transcriptome was again mapped back to the individual raw reads using Bowtie2 (the mapping protocol was followed as mentioned above). eXpress software was again used to generate total counts for individual libraries for further analysis. We followed a normalization strategy, called remove unwanted variation (RUV), that adjusts for nuisance technical effects by performing factor analysis on suitable sets of control genes (e.g., External RNA Control Consortium (ERCC)^37^ manual modified on October 17, 2016). Using the ERCC spike-ins as controls, we considered k = 1 factors of unwanted variation (see^37^ on the choice of k). Both upper quartile normalization and ERCC-spike-in normalization was done using RUVg filtering software^37^ (manual modified on October 17, 2016). We filtered out ambiguous contig variation by requiring more than 5 reads in at least two biological replicates for each contig. This dataset was designated as YO/I/DRESA_filtered transcriptome (Fig. 2).

EdgeR^33^ was used to identify the differentially expressed genes (DEGs) from the YO/I/DRESA_ filtered transcriptome. Generalized linear (GLM) fit model was used to determine the DEGs. The design matrix for transcriptional expression profiles was generated using two factors: “treatment” (IN, DA, DC, DG, RA, RC, RG) and “time” (day0, day1, day3, day7). At each time point, the number of significant DEGs was established by pairwise comparison of libraries: DMSO.1d vs DMSO.3d, DMSO.3d vs DMSO.7d, DMSO.1d vs DMSO.7d, Rapamycin.1d vs Rapamycin.3d, Rapamycin.1d vs Rapamycin.7d, Rapamycin.3d vs Rapamycin.7d, DMSO.1d vs Rapamycin.1d, DMSO.3d vs Rapamycin.3d, DMSO.7d vs Rapamycin.7d, DMSO.1d vs IN.0d, DMSO.3d vs IN.0d, DMSO.7d vs IN.0d, Rapamycin.1d vs IN.0d, Rapamycin.3d vs IN.0d, Rapamycin.7d vs IN.0d. For the details on the design matrix please refer to the R-code in the Suppl. scripts and Suppl. Table 1. For the statistical analysis, relative expression was quantified as a ratio of Log2 (experimental/control). Genes were recognized as differentially expressed (DEG) using the Benjamini-Hochberg method (false discovery rate of <0.05) and log2 fold change <−2 or >+2 implemented by EdgeR (Fig. 2). A detailed script for the DEG analysis using EdgeR is described in the Suppl. scripts. Further data manipulation and statistical analyses (correlation coefficients) were performed using R statistical software (R-Development-Core-Team, 2015).

### Annotation and pathway analysis

For annotation, both nucleotide sequences and predicted peptide sequences were used to run BLAST queries against Swiss-Prot (SP), TrEMBL, and Uniprot Uniref90 protein databases (http://www.uniprot.org/downloads; Fig. 3)^79,80^. SP database was downloaded on September 27, 2016, TrEMBL on October 14, 2016, and Uniref90 on November 28, 2016. Stand-alone software was used for running BLAST (version number: 2.5.0) against the above-mentioned databases (http://blast.ncbi.nlm.nih.gov/Blast.cgi?PAGE_TYPE=BlastDocs&DOC_TYPE=Download). The results from BLASTx against SP and Uniprot databases were in tabular format (output format 6). The e-value cutoff was set at 1e−5 and the first 10 annotation hits per transcript retained.

TransDecoder (version number: v2.0.1; https://transdecoder.github.io/) was used to predict coding peptide sequences from the YO/I/DRESA transcriptome contig sequences. These peptide sequences were annotated via BLASTp against known databases with a cutoff of 1e−5. In order to identify conserved protein families among the predicted peptide sequences, HMMER hmmscan (https://svn.janelia.org/eddylab/eddys/src/hmmer/trunk/documentation/man/hmmscan.man) was used to search for sequences against a Pfam-A database (downloaded on April 19, 2016) (http://hmmer.janelia.org/). In addition, transmembrane helical domains, cleavage sites for signal peptides were identified using TMHMM (version number: 2.0c), and SignalP (version number: 4.1) software respectively. All the outputs obtained from BLAST, HMMER, TMHMM and SignalP were used to achieve a comprehensive annotation for each contig. We used Trinotate (version number: v3.0.2) to generate a flat file report containing all annotation information for each contig^30^ (Fig. 3).

Due to the lack of a well annotated genome, BLASTx was run against the protein databases from human (*Homo sapiens*) and 6 phylogenetically conserved insect species to generate a transcriptome database specific to crustaceans designated as: KO_insect-specific.fasta. The six insect species used were *Tribolium castaneum*, *Bombyx mori*, *Ixodes scapularis*, *Nasonia vitripennis*, *Drosophila melanogaster*, and *Harpegnathos saltator*. We used regularly updated auxiliary mapping files for KO and pathway assignments rather than older built-in KEGG pathways (http://www.genome.jp/kegg/pathway.html). KO_insect-specific.fasta was blasted against the KEGG annotations to the Kyoto Encyclopedia for Genes and Genomes (KEGG) pathways and KEGG orthology (KO) (http://www.genome.jp/kegg/ko.html) databases. The KEGG Automatic Annotation Server (KAAS) software (http://www.genome.jp/tools/kaas/) was used to obtain KO numbers to create a summary of the KO terms associated with annotated transcripts in the YO/I/DRESA_filtered transcriptome (Fig. 3).

The annotated transcriptome in Excel format is available in the Cyverse Discovery environment at: https://de.cyverse.org/dl/d/7454F0B2-1E3D-4A27-A901-17A0D6BA8CDE/ESA_annotation_report_1stNov16.xls.

### Computational resources

The assembly of the YO/I/DRESA transcriptome was performed at the Oklahoma State University High Performance Computing Center using SSH (http://hpc.it.okstate.edu/). Computations required for preparation of assembly, steady-state abundance estimations and annotation were performed on local departmental computing resources. Protocols and command line programming used for pipeline integration are described in the Suppl. scripts.

## Electronic supplementary material

Supplementary Figures and Table

## References

[1] Shyamal S, Sudha K, Gayathri N, Anilkumar G (2014). The Y-organ secretory activity fluctuates in relation to seasons of molt and reproduction in the brachyuran crab, *Metopograpsus messor* (Grapsidae): Ultrastructural and immunohistochemical study. Gen. Comp. Endocrinol..

[2] Skinner, D.M. Molting and regeneration in *The Biology of Crustacea*, (ed. Bliss, D. E. & Mantel, L. H.), 43–146 (Academic Press 1985).

[3] Webster SG, Keller R, Dircksen H (2012). The CHH-superfamily of multifunctional peptide hormones controlling crustacean metabolism, osmoregulation, moulting, and reproduction. Gen. Comp. Endocrinol..

[4] Webster, S.G. Endocrinology of molting in *The Natural History of Crustacea: Physiology*, 1–35 (Oxford University Press 2015).

[5] Chang ES, Mykles DL (2011). Regulation of crustacean molting: A review and our perspectives. Gen. Comp. Endocrinol..

[6] Hopkins PM (2012). The eyes have it: A brief history of crustacean neuroendocrinology. Gen. Comp. Endocrinol..

[7] Nakatsuji T, Lee C-Y, Watson RD (2009). Crustacean molt-inhibiting hormone: structure, function, and cellular mode of action. Comp. Biochem. Physiol. A.

[8] Chung JS, Zmora N, Katayama H, Tsutsui N (2010). Crustacean hyperglycemic hormone (CHH) neuropeptides family: Functions, titer, and binding to target tissues. Gen. Comp. Endocrinol..

[9] Mykles DL (2001). Interactions between limb regeneration and molting in decapod crustaceans. Am. Zool..

[10] Yu X, Chang ES, Mykles DL (2002). Characterization of Limb Autotomy Factor-proecdysis (LAFpro), isolated from limb regenerates, that suspends molting in the land crab *Gecarcinus lateralis*. Biol. Bull..

[11] Covi JA, Chang ES, Mykles DL (2012). Neuropeptide signaling mechanisms in crustacean and insect molting glands. Invertebr. Repr. Dev..

[12] Covi JA, Chang ES, Mykles DL (2009). Conserved role of cyclic nucleotides in the regulation of ecdysteroidogenesis by the crustacean molting gland. Comp. Biochem. Physiol. A.

[13] Abuhagr AM, MacLea KS, Chang ES, Mykles DL (2014). Mechanistic target of rapamycin (mTOR) signaling genes in decapod crustaceans: cloning and tissue expression of mTOR, Akt, Rheb, and p70 S6 kinase in the green crab, *Carcinus maenas*, and blackback land crab, *Gecarcinus lateralis*. Comp. Biochem. Physiol. A.

[14] Abuhagr AM (2016). Roles of mechanistic target of rapamycin and transforming growth factor-β signaling in the molting gland (Y-organ) of the blackback land crab, *Gecarcinus lateralis*. Comp. Biochem. Physiol. A.

[15] Baretic D, Williams RL (2014). The structural basis for mTOR function. Sem. Cell Dev. Biol..

[16] Nandagopal N, Roux PP (2015). Regulation of global and specific mRNA translation by the mTOR signaling pathway. Translation (Austin).

[17] Saxton RA, Sabatini DM (2017). mTOR Signaling in Growth, Metabolism, and Disease. Cell.

[18] Frias MA (2006). mSin1 is necessary for Akt/PKB phosphorylation, and its isoforms define three distinct mTORC2s. Curr. Biol..

[19] Laplante M, Sabatini DM (2012). mTOR signaling in growth control and disease. Cell.

[20] Inoki K, Li Y, Zhu T, Wu J, Guan K-L (2002). TSC2 is phosphorylated and inhibited by Akt and suppresses mTOR signaling. Nat. Cell Biol..

[21] Huang J, Manning BD (2009). A complex interplay between Akt, TSC2, and the two mTOR complexes. Biochem. Soc. Trans..

[22] Zarogoulidis P (2014). mTOR pathway: A current, up-to-date mini-review (Review). Oncol. Lett..

[23] Pentek J, Parker L, Wu A, Arora K (2009). Follistatin preferentially antagonizes activin rather than BMP signaling in *Drosophila*. Genesis.

[24] Gibbens YY, Warren JT, Gilbert LI, O’Connor MB (2011). Neuroendocrine regulation of *Drosophila* metamorphosis requires TGFβ/Activin signaling. Development.

[25] Rewitz KF, Yamanaka N, O’Connor MB (2013). Developmental checkpoints and feedback circuits time insect maturation. Curr. Top. Dev. Biol..

[26] Yamanaka N, Rewitz KF, O’Connor MB (2013). Ecdysone control of developmental transitions: lessons from *Drosophila* research. Annu. Rev. Entomol..

[27] Ishimaru Y (2016). TGF-β signaling in insects regulates metamorphosis via juvenile hormone biosynthesis. PNAS.

[28] Wang Z, Gerstein M, Snyder M (2009). RNA-Seq: a revolutionary tool for transcriptomics. Nat. Rev. Genet..

[29] Das S, Pitts NL, Mudron MR, Durica DS, Mykles DL (2016). Transcriptome analysis of the molting gland (Y-organ) from the blackback land crab, *Gecarcinus lateralis*. Comp. Biochem. Physiol. D.

[30] Haas BJ (2013). *De novo* transcript sequence reconstruction from RNA-seq using the Trinity platform for reference generation and analysis. Nat. Protoc..

[31] Li W, Godzik A (2006). Cd-hit: a fast program for clustering and comparing large sets of protein or nucleotide sequences. Bioinformatics.

[32] Langmead B, Salzberg SL (2012). Fast gapped-read alignment with Bowtie 2. Nat. Meth..

[33] Robinson MD, McCarthy DJ, Smyth GK (2010). edgeR: a Bioconductor package for differential expression analysis of digital gene expression data. Bioinformatics.

[34] Ghaffari, N. *et al*. Novel transcriptome assembly and improved annotation of the whiteleg shrimp (*Litopenaeus vannamei*), a dominant crustacean in global seafood mariculture. *Sci*. *Rep*. **4**, 10.1038/srep07081 (2014). 10.1038/srep07081 PMC4243063 25420880

[35] Lenz PH (2014). *De novo* assembly of a transcriptome for *Calanus finmarchicus* (Crustacea, Copepoda) – the dominant zooplankter of the North Atlantic Ocean. Plos One.

[36] Armstrong EJ, Stillman JH (2016). Construction and characterization of two novel transcriptome assemblies in the congeneric porcelain crabs *Petrolisthes cinctipes* and *P*. *manimaculis*. Integr. Comp. Biol..

[37] Risso D, Ngai J, Speed TP, Dudoit S (2014). Normalization of RNA-seq data using factor analysis of control genes or samples. Nat. Biotechnol..

[38] Moriya, Y. *et al*. KAAS: an automatic genome annotation and pathway reconstruction server. *Nucl*. *Acids Res*. **35**, 10.1093/nar/gkm321 (2007). 10.1093/nar/gkm321 PMC1933193 17526522

[39] Kanehisa M, Goto S (2000). KEGG: Kyoto Encyclopedia of Genes and Genomes. Nucleic Acids Res..

[40] Kanehisa M, Sato Y, Kawashima M, Furumichi M, Tanabe M (2016). KEGG as a reference resource for gene and protein annotation. Nucleic Acids Res..

[41] Kanehisa Furumichi, M., Tanabe, M., Sato, Y. & Morishima, K. KEGG: new perspectives on genomes, pathways, diseases and drugs. **45**, D353–D361 (2017). 10.1093/nar/gkw1092 PMC5210567 27899662

[42] Tom M, Manfrin C, Giulianini PG, Pallavicini A (2013). Crustacean oxi-reductases protein sequences derived from a functional genomic project potentially involved in ecdysteroid hormones metabolism – A starting point for function examination. Gen. Comp. Endocrinol..

[43] Mykles DL (2011). Ecdysteroid metabolism in crustaceans. J. Steroid Biochem. Mol. Biol..

[44] Xie X (2015). Hemolymph Levels of Methyl Farnesoate During Ovarian Development of the Swimming Crab *Portunus trituberculatus*, and Its Relation to Transcript Levels of HMG-CoA Reductase and Farnesoic Acid O-Methyltransferase. Biol. Bull..

[45] Pitts NL, Schulz HM, Oatman SR, Mykles DL (2017). Elevated expression of neuropeptide signaling genes in the eyestalk ganglia and Y-organ of *Gecarcinus lateralis* individuals that are refractory to molt induction. Comp. Biochem. Physiol. A.

[46] Kim D-H (2002). mTOR interacts with raptor to form a nutrient-sensitive complex that signals to the cell growth machinery. Cell.

[47] Rodriguez Camargo DC, Link NM, Dames SA (2012). The FKBP–rapamycin binding domain of human TOR undergoes strong conformational changes in the presence of membrane mimetics with and without the regulator phosphatidic acid. Biochemistry.

[48] März AM, Fabian A-K, Kozany C, Bracher A, Hausch F (2013). Large FK506-binding proteins shape the pharmacology of rapamycin. Mol. Cell. Biol..

[49] Sarbassov DD (2006). Prolonged rapamycin treatment inhibits mTORC2 assembly and Akt/PKB. Mol. Cell.

[50] Betz C, Hall MN (2013). Where is mTOR and what is it doing there?. J. Cell Biol..

[51] Su B, Jacinto E (2011). Mammalian TOR signaling to the AGC kinases. Crit. Rev. Biochem. Mol. Biol..

[52] Yang H (2013). mTOR kinase structure, mechanism and regulation by the rapamycin-binding domain. Nature.

[53] Magnuson B, Ekim B, Fingar DC (2012). Regulation and function of ribosomal protein S6 kinase (S6K) within mTOR signalling networks. Biochem. J..

[54] Hall DJ, Grewal SS, de la Cruz AFA, Edgar BA (2007). Rheb-TOR signaling promotes protein synthesis, but not glucose or amino acid import, in *Drosophila*. BMC Biol..

[55] Gu S-H, Yeh W-L, Young S-C, Lin P-L, Li S (2012). TOR signaling is involved in PTTH-stimulated ecdysteroidogenesis by prothoracic glands in the silkworm, *Bombyx mori*. Insect Biochem. Mol. Biol..

[56] Kemirembe K (2012). Amino acids and TOR signaling promote prothoracic gland growth and the initiation of larval molts in the tobacco hornworm *Manduca sexta*. Plos One.

[57] Danielsen ET (2016). A *Drosophila* genome-wide screen identifies regulators of steroid hormone production and developmental timing. Dev. Cell.

[58] Schmitt S, Ugrankar R, Greene SE, Prajapati M, Lehmann M (2015). *Drosophila* Lipin interacts with insulin and TOR signaling pathways in the control of growth and lipid metabolism. J. Cell Sci..

[59] Parker J, Struhl G (2015). Scaling the *Drosophila* wing: TOR-dependent target gene access by the hippo pathway transducer yorkie. Plos Biol..

[60] Varma D, Bülow MH, Pesch Y-Y, Loch G, Hoch M (2014). Forkhead, a new cross regulator of metabolism and innate immunity downstream of TOR in *Drosophila*. J. Insect Physiol..

[61] Marshall L, Rideout EJ, Grewal SS (2012). Nutrient/TOR-dependent regulation of RNA polymerase III controls tissue and organismal growth in *Drosophila*. EMBO J..

[62] MacLea KS (2012). Rheb, an activator of target of rapamycin, in the blackback land crab, *Gecarcinus lateralis*: cloning and effects of molting and unweighting on expression in skeletal muscle. J. Exp. Biol..

[63] Vasudevan K. M. & Garraway L. A. AKT Signaling in Physiology and Disease in *Phosphoinositide 3-kinase in Health and Disease* (ed. Rommel, C., Vanhaesebroeck, B. & Vogt, P.) *Curr*. *Top*. *Microbiol*. *Immunol*. **347**, 105–133. (Springer 2010). 10.1007/82_2010_66 20549472

[64] Aspuria P-J, Tamanoi F (2004). The Rheb family of GTP-binding proteins. Cell. Signal..

[65] Frost RA, Lang CH (2011). mTOR signaling in skeletal muscle during sepsis and inflammation: Where does it all go wrong?. Physiology.

[66] Brattström, C. *et al*. Kinetics and dynamics of single oral doses of sirolimus in sixteen renal transplant recipients. *Ther*. *Drug Monit*. **19**, 397–406 (1997). 10.1097/00007691-199708000-00007 9263380

[67] Singh K (2014). Superiority of rapamycin over tacrolimus in preserving non-human primate Treg half-life and phenotype after adoptive transfer. Am. J. Transplant..

[68] Xie X (2016). Role of Halloween genes in ecdysteroids biosynthesis of the swimming crab (*Portunus trituberculatus*): Implications from RNA interference and eyestalk ablation. Comp. Biochem. Physiol. A: Mol. Integr. Physiol..

[69] Komura-Kawa T (2015). The *Drosophila* zinc finger transcription factor Ouija board controls ecdysteroid biosynthesis through specific regulation of spookier. PLOS Genetics.

[70] Niwa YS, Niwa R (2016). Ouija board: A transcription factor evolved for only one target in steroid hormone biosynthesis in the fruit fly *Drosophila melanogaster*. Transcription.

[71] Niwa YS, Niwa R (2016). Transcriptional regulation of insect steroid hormone biosynthesis and its role in controlling timing of molting and metamorphosis. Develop. Growth Differ..

[72] Danielsen ET (2014). Transcriptional Control of Steroid Biosynthesis Genes in the Drosophila Prothoracic Gland by Ventral Veins Lacking and Knirps. Plos Genetics.

[73] Alexandratos A, Nellas I, Mavridis K, Moulos P, Dedos SG (2016). Reassessing ecdysteroidogenic cells from the cell membrane receptors’ perspective. Sci. Rep..

[74] Switon K, Kotulska K, Janusz-Kaminska A, Zmorzynska J, Jaworski J (2017). Molecular neurobiology of mTOR. Neuroscience.

[75] Abuhagr AM (2014). Molt regulation in green and red color morphs of the crab *Carcinus maenas*: gene expression of molt-inhibiting hormone signaling components. J. Exp. Biol..

[76] Kingan T (1989). A competitive enzyme-linked immunosorbent assay: applications in the assay of peptides, steroids, and cyclic nucleotides. Anal. Biochem..

[77] Bolger AM, Lohse M, Usadel B (2014). Trimmomatic: a flexible trimmer for Illumina sequence data. Bioinformatics.

[78] Roberts A, Pachter L (2013). Streaming fragment assignment for real-time analysis of sequencing experiments. Nat. Methods.

[79] Altschul SF (1997). Gapped BLAST and PSI-BLAST: a new generation of protein database search programs. Nucleic Acids Res..

[80] Bairoch A, Apweiler R (2000). The SWISS-PROT protein sequence database and its supplement TrEMBL in 2000. Nucleic Acids Res..

