# Supplementary material for: Transcriptomic analysis of crustacean molting gland (Y-organ) regulation via the mTOR signaling pathway
Source: Sci Rep. 2018 May 9;8:7307. doi: 10.1038/s41598-018-25368-x (PMC5943448; doi:10.1038/s41598-018-25368-x)
Supplement: Supplementary file 1 — Supplementary Figures and Table [file 41598_2018_25368_MOESM1_ESM.pdf]

Transcriptomic analysis of crustacean molting gland (Y-organ) regulation via the mTOR signaling pathway

Shyamal S.[1], Das S.[2], Guruacharya A.[1], Mykles D.L.[2], and Durica D.S.[1]*

[1]Department of Biology, University of Oklahoma, Norman, Oklahoma, 73019, USA; [2]Department of Biology, Colorado State University, Fort Collins, Colorado, 80523, USA

*Corresponding author: ddurica@ou.edu

Supplementary Figures

A.

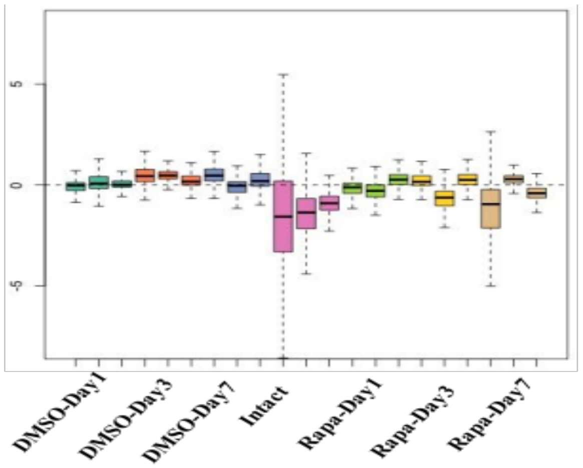

B.

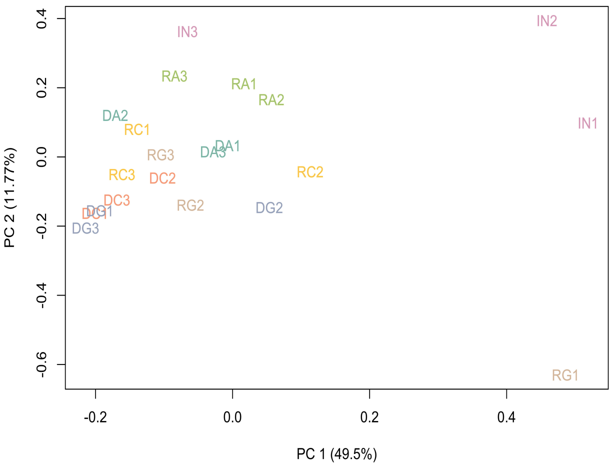

C.

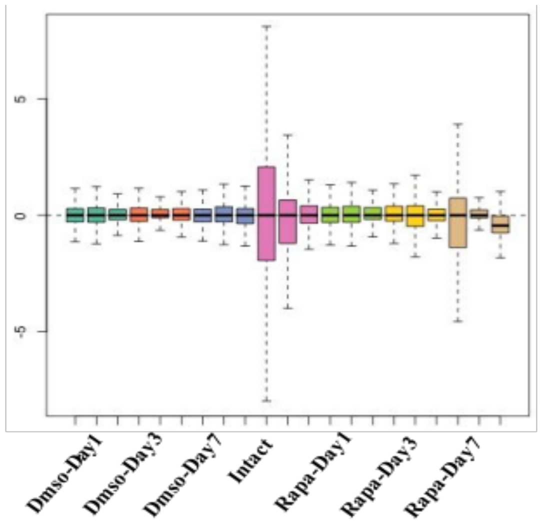

D.

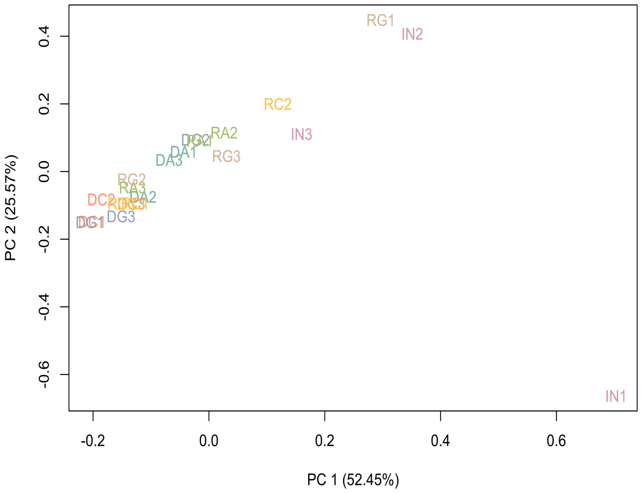

E.

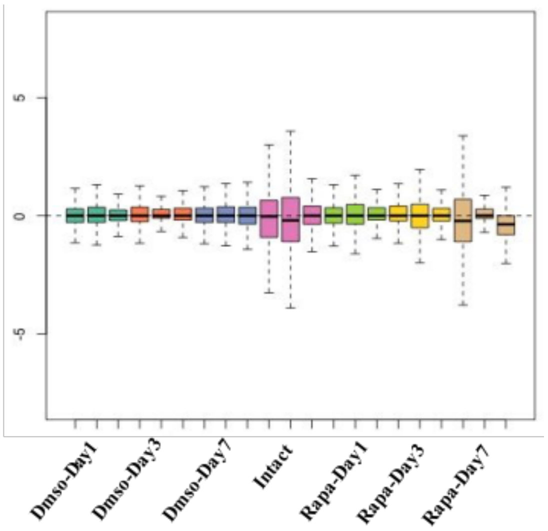

F.

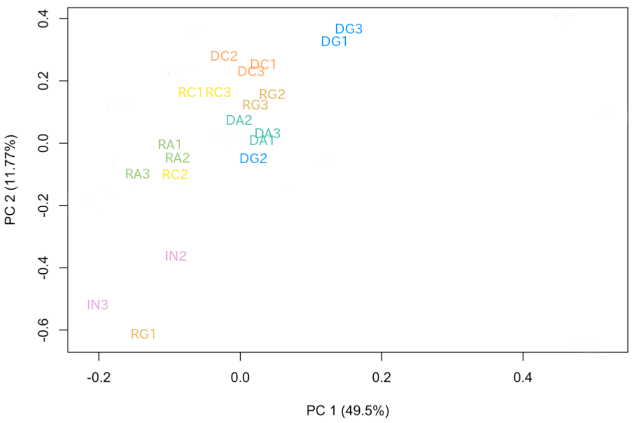

Supplementary Figure 1. Removal of unwanted variation in the YO RNA-seq data set. We expect relative log expression distributions (RLE) to be centered around zero and similar to each other. (1A) The RLE boxplots prior to normalization. (The bottom and top of the box indicate, respectively, the first and third quartiles; the inside line indicates the median. (1B) Scatterplot of first two principal components for un-normalized counts (log scale, centered). Libraries do not cluster as expected according to treatment. (1C) Upper quartile normalization centers RLE around zero, but replicate interlibrary variability is high. (1D) Scatterplot of first two principal components for upper quartile normalization. (1E) Boxplots of RLE for RUVg-normalized counts. RUVg based on spike-ins leads to more uniform RLE distributions. (1F) Libraries cluster as expected by treatment; note IN1 (intact/intermolt replicate library 1) outlier (IN1 PC1/2 value 1.473). Pearson correlation coefficients are given in table 3. Library designations are IN: intact/intermolt libraries; D: DMSO injection libraries following ESA; R: rapamycin injection libraries following ESA. All libraries had 3 technical replicates (1-3). D and R libraries were taken following day 1 (A) day 3 (C) and day 7 (G) following ESA.

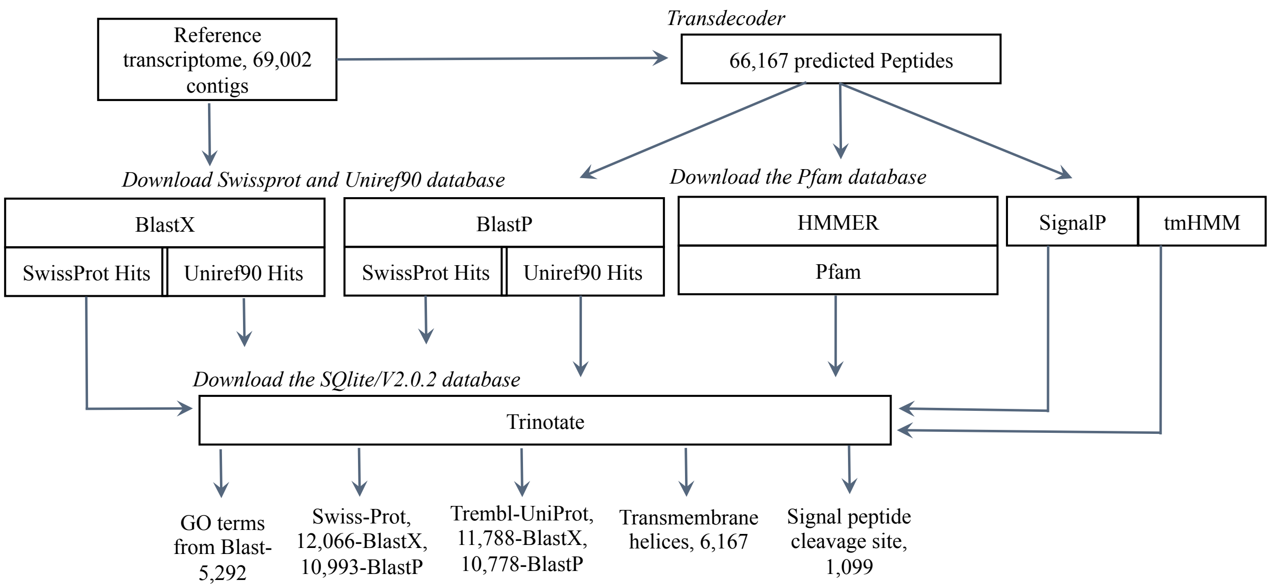

Supplementary Figure 2. Annotation pipeline. Both nucleotide and peptide sequences obtained from the reference YO transcriptome were used for BLAST analyses against four databases. Using Trinotate, GO IDs with associated gene and domain identifiers were extracted. GO Slim Viewer was used to generate a summary of functions and GO Slim IDs (see text for details). The downloaded databases are indicated in italics; numbers represent contig assignments resulting from searches.

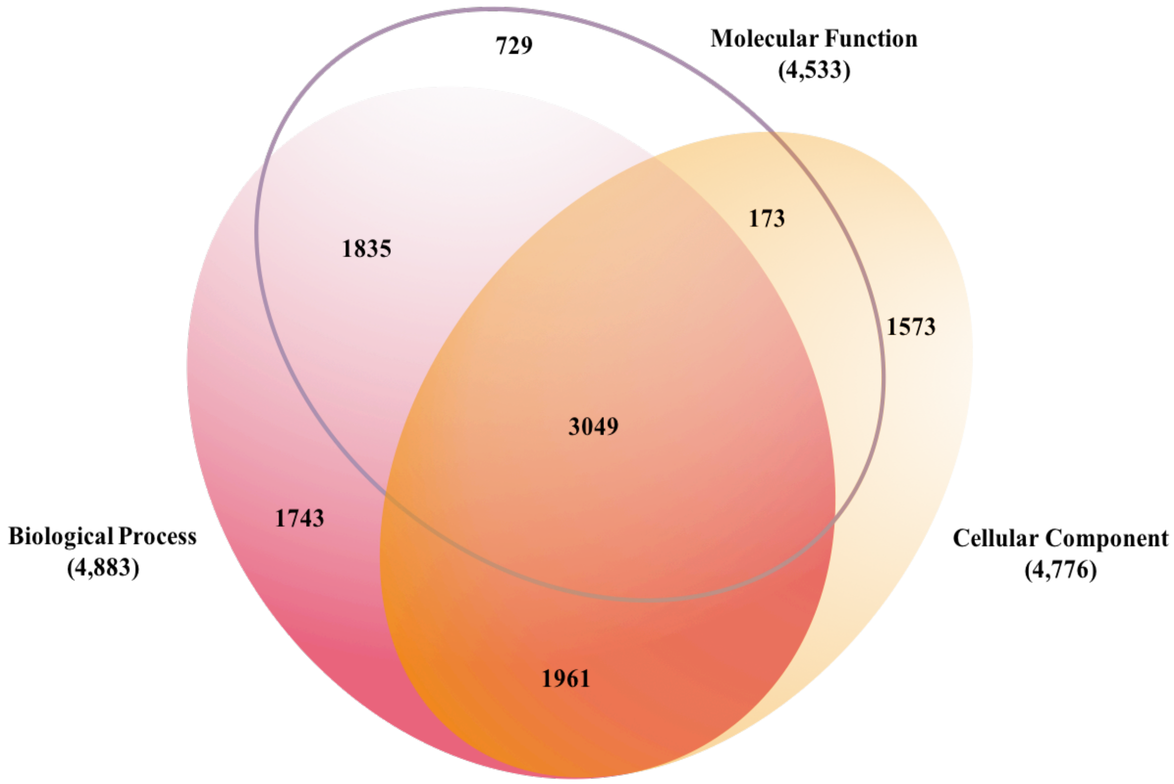

Supplementary Figure 3. Venn diagram illustrates the distribution of unique contigs annotated to one or combination of the three GO categories (Suppl. Fig. 4). Many of the contigs were assigned to two or all three categories.

A. Molecular Function

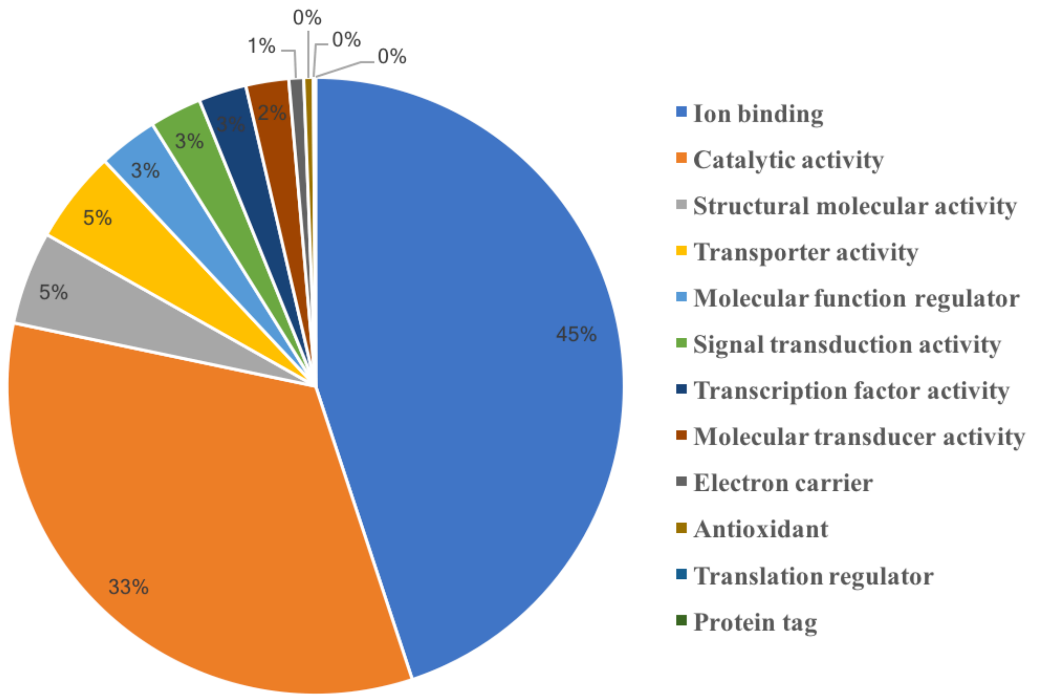

B. Cellular Component

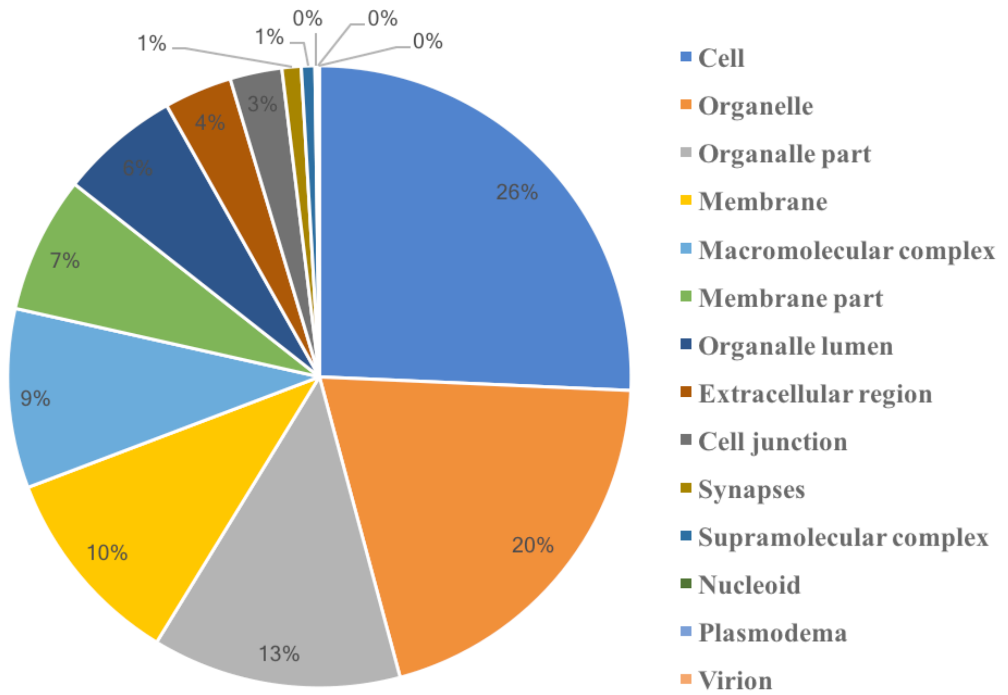

C. Biological Processes

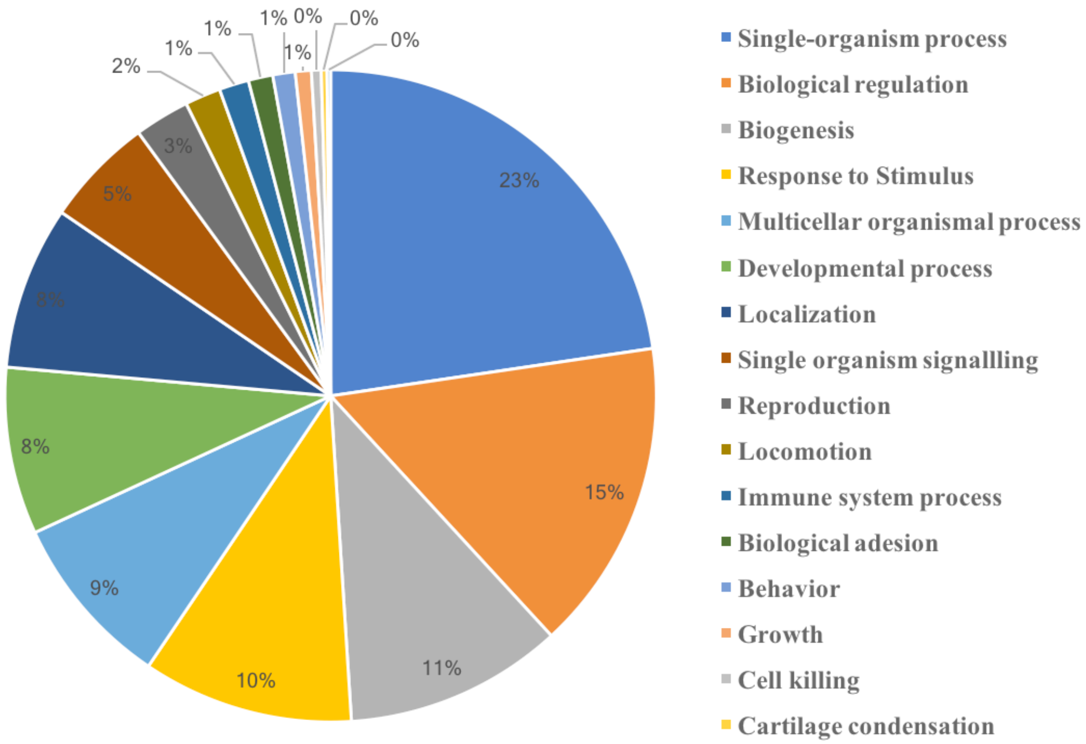

Supplementary Figure 4. Number of reference transcriptome contigs distributed among second-tier GO terms. The sections show the distribution of second-tier GO terms associated with three parent terms: molecular function (A), cellular component (B), and biological process (C). The sections rank the terms from the highest to lowest number of contigs in each of the second-tier GO categories.

A. Heat map showing the log2fold change of the FPKM values of the DEGs representing the mTOR signaling pathway

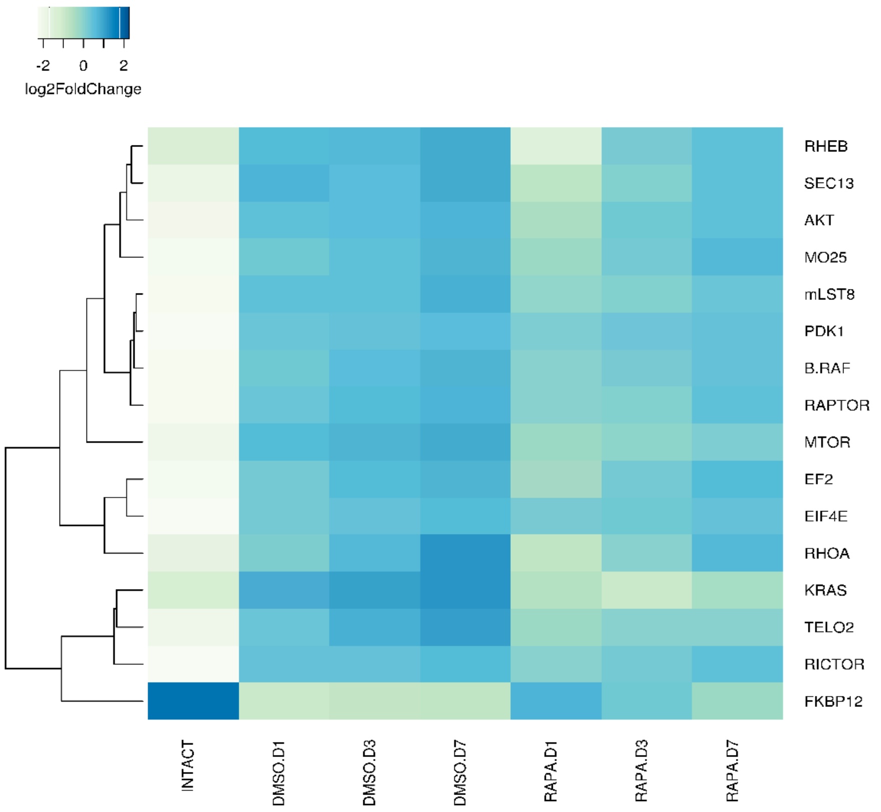

B. Changes in mRNA abundance for mTOR signaling pathway genes

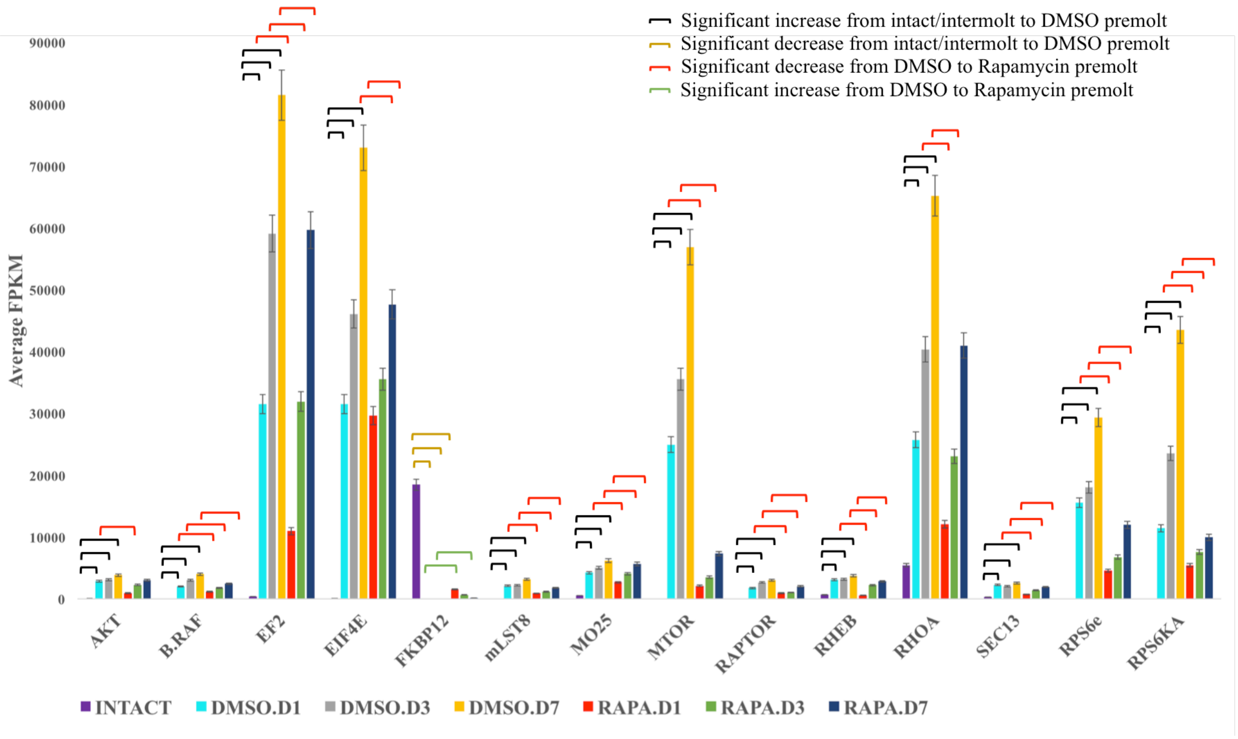

C. Heat map showing the log2fold change of the FPKM values of the DEGs representing the AMPK signaling pathway

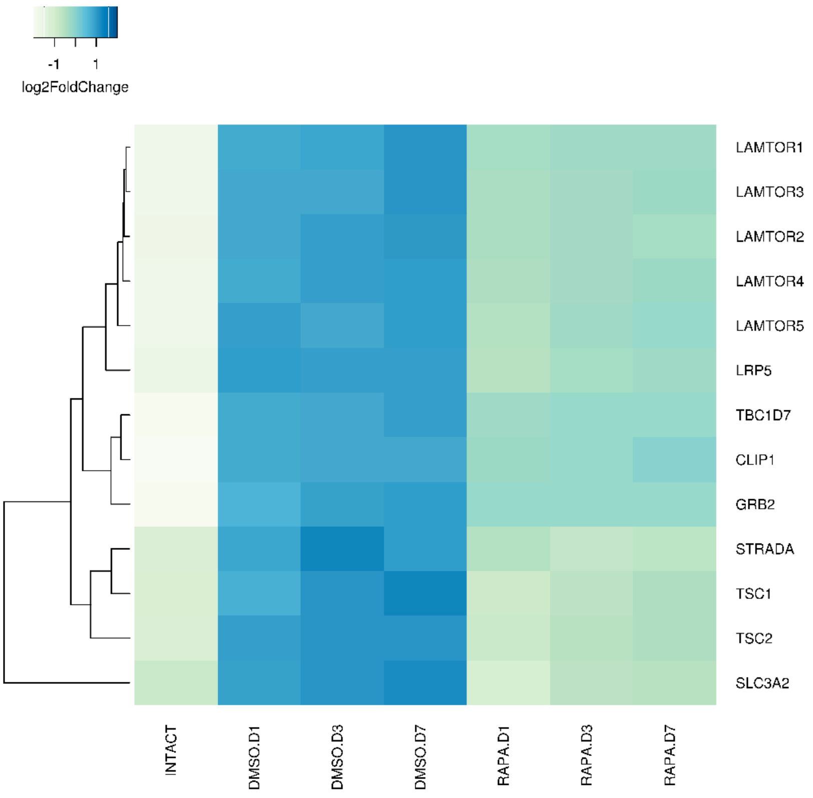

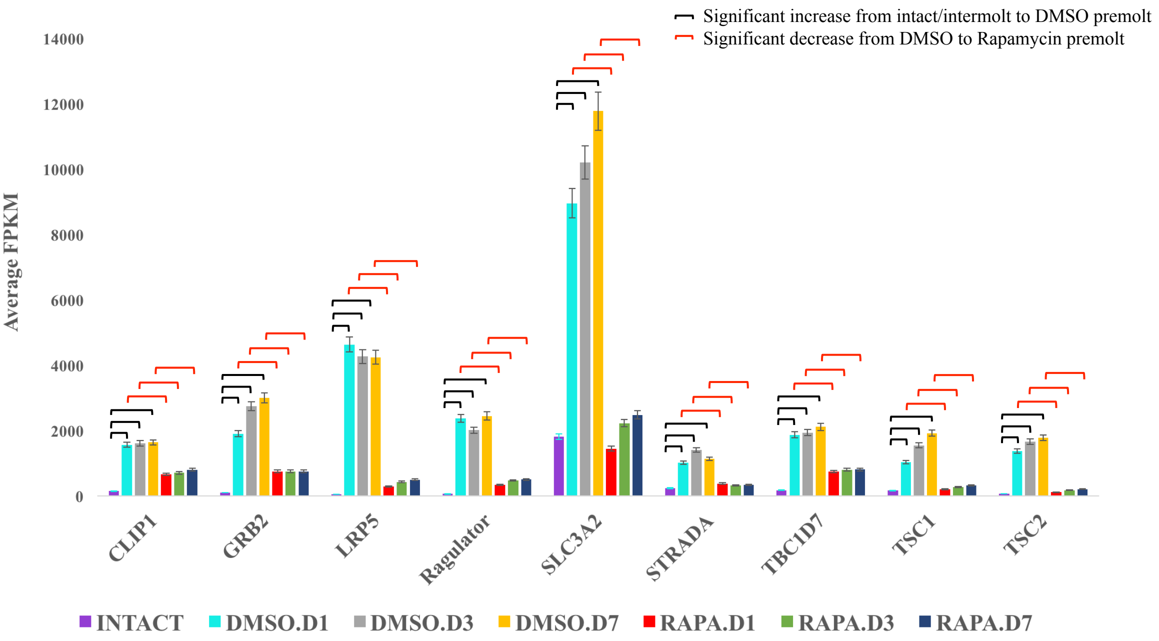

E. Heat map showing the log2fold change of the FPKM values of the DEGs representing the Wnt signaling pathway

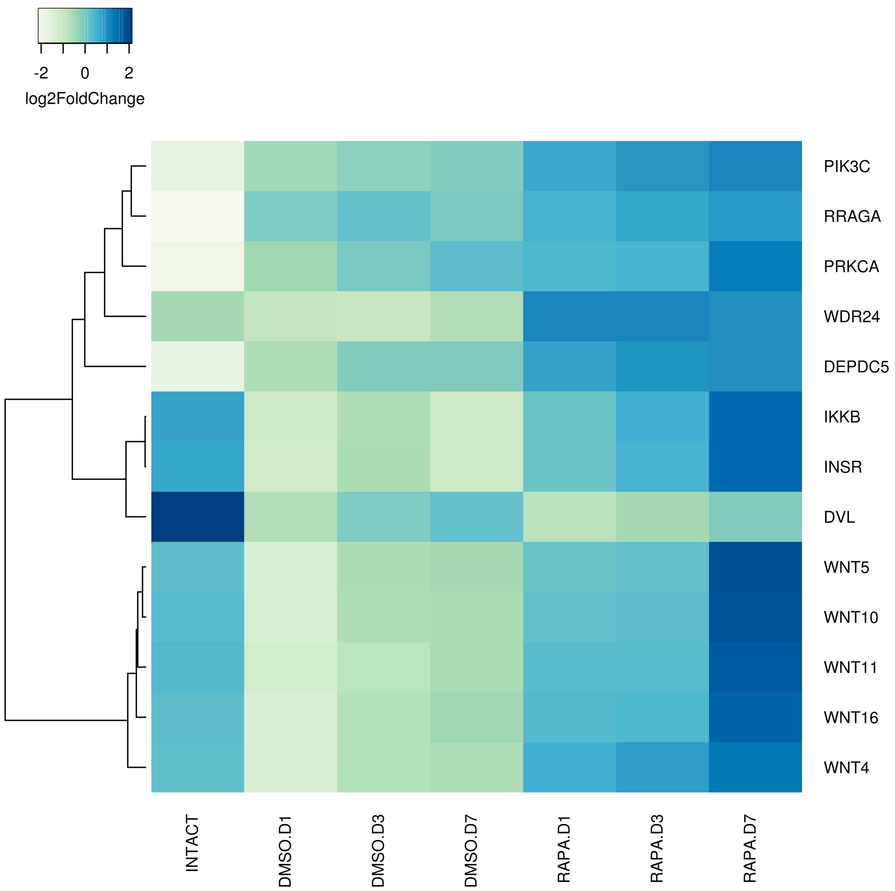

F. Changes in mRNA abundance for Wnt signaling pathway genes.

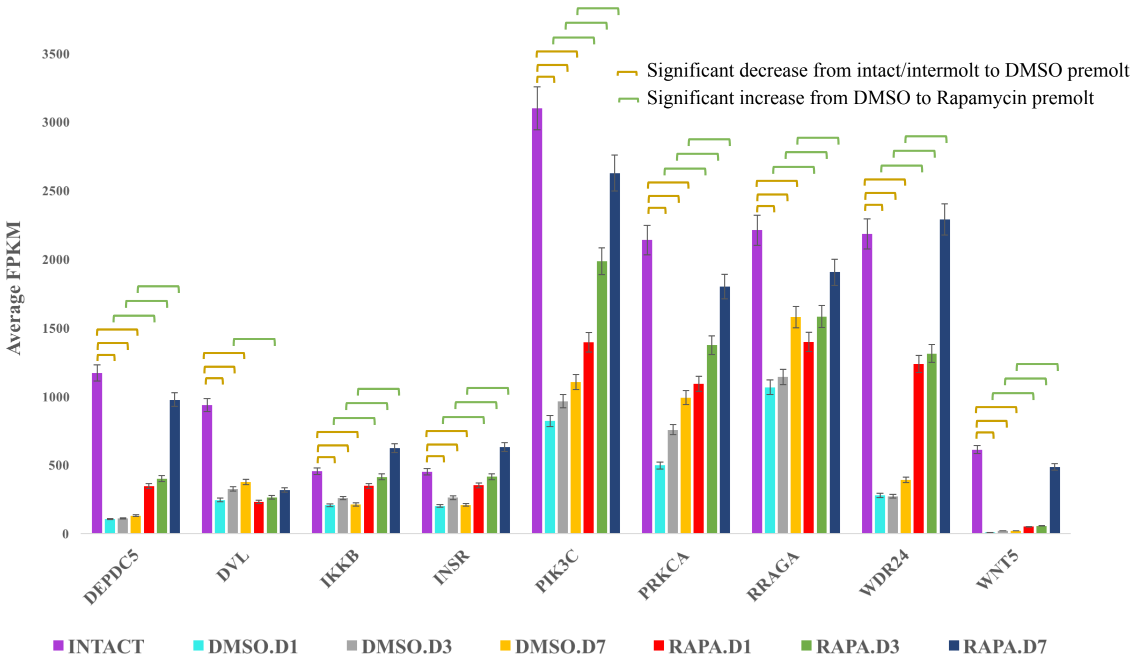

G. Changes in mRNA abundance for insect hormone biosynthetic pathway genes

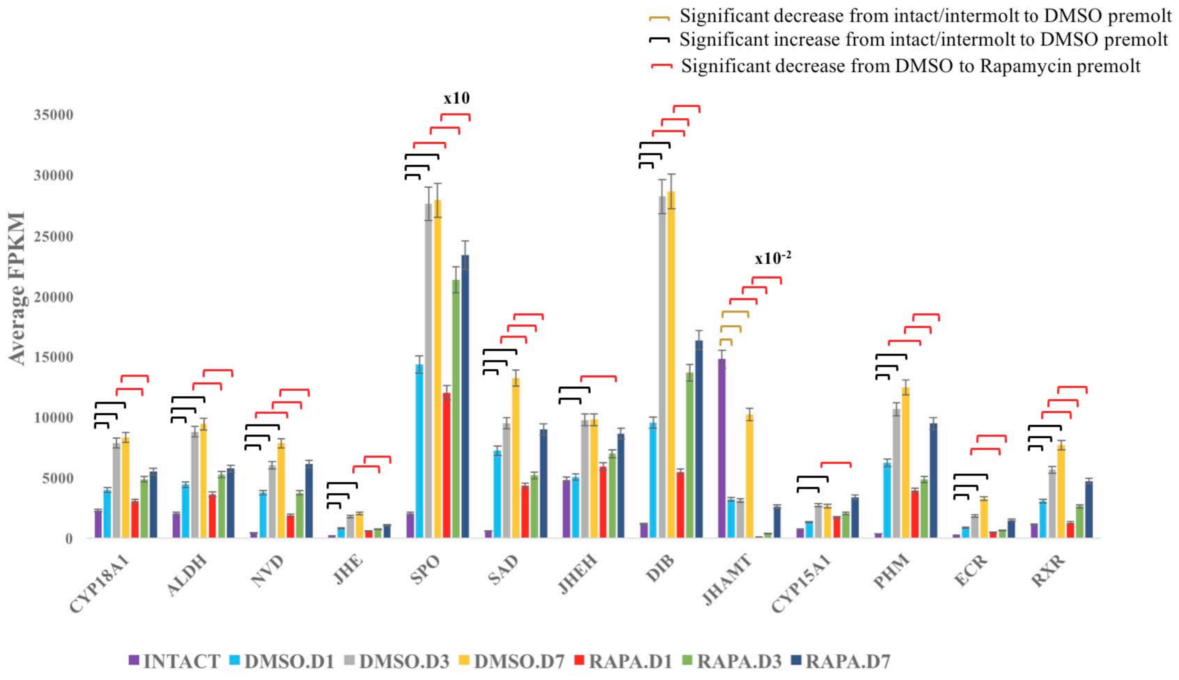

H. Changes in mRNA abundance for Molt Inhibiting Hormone pathway genes

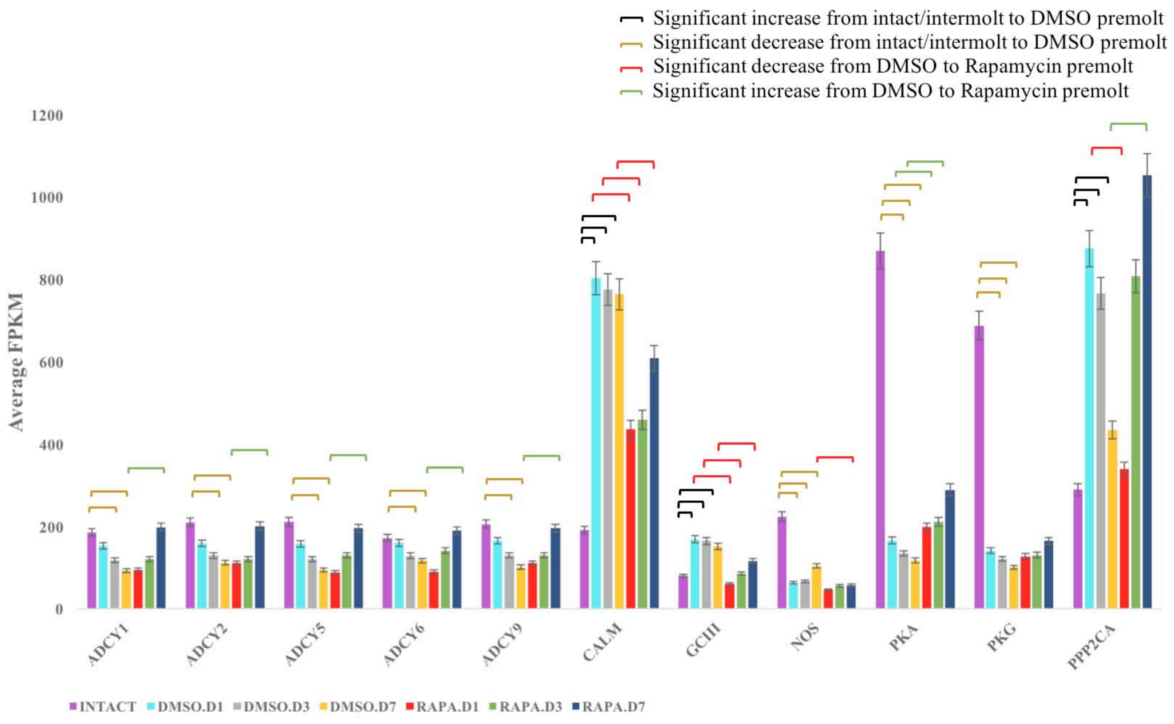

I. Changes in mRNA abundance for TGFβ pathway genes

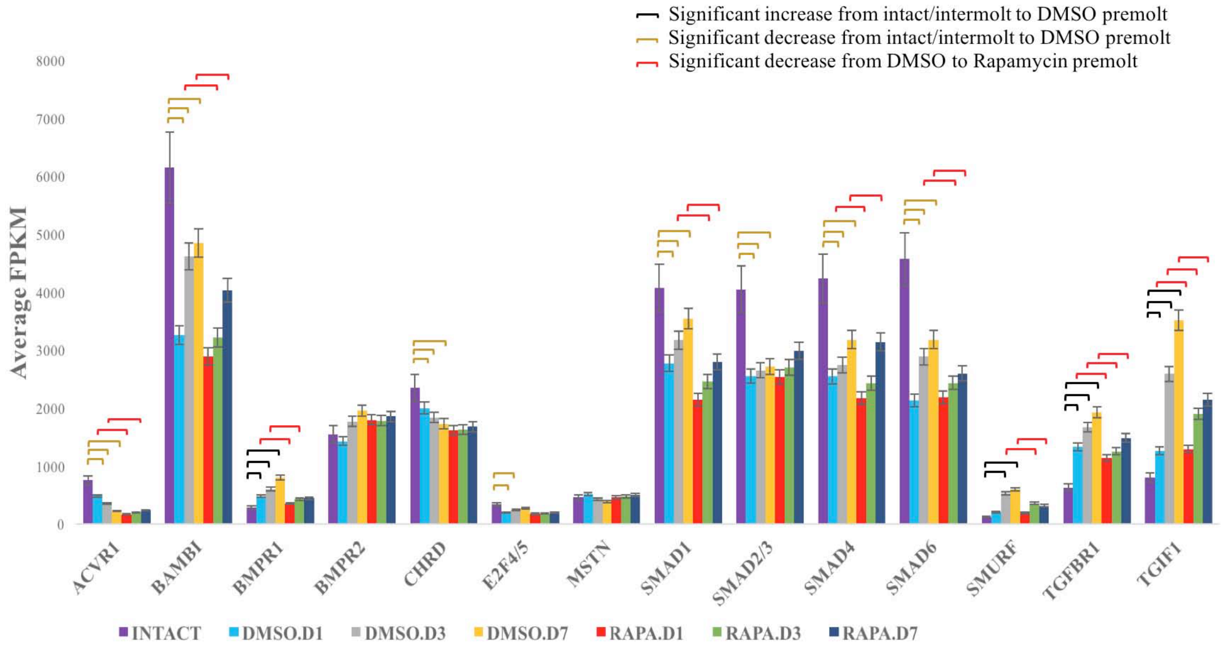

Supplementary Figure 5. Graphical representation of expression levels of selected components from four signaling pathways: mTOR/WNT/AMPK/MIH/TGFβ (A) Heat map for mTOR DEGs; (B) Histogram identifying significant differences in average FPKM values across libraries for mTOR pathway genes; (C) Heat map for AMPK DEGs; (D) Histogram identifying significant differences in average FPKM values across libraries for AMPK pathway genes; (E) Heat map for Wnt DEGs; (F) Histogram identifying significant differences in average FPKM values across libraries for Wnt pathway genes; (G) Histogram identifying significant differences in average FPKM values across libraries for insect hormone biosynthetic pathway genes (note scale change for SPO [10x increase] and JHAMT [100x decrease]); (H) Histogram identifying significant differences in average FPKM values across libraries for Molt Inhibiting Hormone pathway genes; (I) Histogram identifying significant differences in average FPKM values across libraries for TGFβ pathway genes. The differentially expressed genes were identified via ANOVA. Statistical significance was detected at 0.05 p-value. A total of 464 genes were selected from these four pathways, of which 106 were differentially expressed across the molt cycle.

	**Treatment**	**Time**
**Sample 1**	IN	0d
**Sample 2**	IN	0d
**Sample 3**	DMSO	1d
**Sample 4**	DMSO	1d
**Sample 5**	DMSO	1d
**Sample 6**	Rapamycin	1d
**Sample 7**	Rapamycin	1d
**Sample 8**	Rapamycin	1d
**Sample 9**	DMSO	3d
**Sample 10**	DMSO	3d
**Sample 11**	DMSO	3d
**Sample 12**	Rapamycin	3d
**Sample 13**	Rapamycin	3d
**Sample 14**	Rapamycin	3d
**Sample 15**	DMSO	7d
**Sample 16**	DMSO	7d
**Sample 17**	DMSO	7d
**Sample 18**	Rapamycin	7d
**Sample 19**	Rapamycin	7d
**Sample 20**	Rapamycin	7d

Supplementary Table 1: Design matrix for the generalized linear (GLM) fit model used for DEG analysis (see text, section 4.4). note IN1 (intact/ intermolt replicate library 1)

<u>Supplementary Scripts</u>

Protocols and command line

All computational jobs were performed on a local departmental computer unless otherwise mentioned in text, section 4.6.

Quality check using fastQC
fastqc ./*.fq.gz

De novo Assembly using Trinity

Trinity --seqType fq --max_memory 180G \
--left ESA_forward_paired.fq.gz \
--right ESA_reverse_paired.fq.gz \
--CPU 12 --output ESA_Trinity.fasta --full_cleanup

Running cdHIT on the trinity output for clustering the files

time cd-hit -T 12 -i ESA_Trinity.fasta -o ESA_99-Cdhit.fasta -c 0.99 -n 5 -M 30000 -d 0 -p 1

Assembly quality check assesment

/opt/trinity/2.0.6/gcc/util/TrinityStats.pl ESA_reference.fasta

Bowtie2 build indexer

bowtie2-build ESA_reference_09_20_16.fasta ESA_ref.bt2

Running bowtie 2

bowtie2 -p 12 --time -x ESA_ref.bt2 -1 ESA_FR_paired.fq.gz -2 ESA_RV_reverse.fq.gz -S ESA.sam.gz

Converting sam fle into bam files by running Samtools

samtools view -b -S -o ESA.bam ESA.sam.gz

Running express to get the count matrix

./express --rf-stranded -o ESA_reference_09_20_16.fasta ESA.bam

Running transdecoder

/opt/transdecoder/2.0.1/gcc/TransDecoder.LongOrfs -t
/media/sharmishtha/LimbBudSharmi/LB-NGS/assembled_LB/LB_ref98_CD-06-01.fasta

Downloaded SwissProt and pfam Database on May 11 2016

SwissProt Download SwissProt here: https:
//data.broadinstitute.org/Trinity/Trinotate_v3_RESOURCES/uniprot_sprot.pep.gz
gunzip uniprot_sprot.pep.gz
makeblastdb -in uniprot_sprot.pep -dbtype prot

Pfam domains Download Pfam-A here: https:
//data.broadinstitute.org/Trinity/Trinotate_v3_RESOURCES/Pfam-A.hmm.gz
gunzip Pfam-A.hmm.gz
hmmpress Pfam-A.hmm

Downloaded Uniref90 Database on Sep 28 2016

wget
https://data.broadinstitute.org/Trinity/Trinotate_v2.0_RESOURCES/uniprot_uniref90.trinotate_v2.0.pep.gz
gzip -d uniprot_uniref90.trinotate_v2.0.pep.gz # unzipping the the database
makeblastdb -in uniprot_uniref90.trinotate_v2.0.pep.gz -parse_seqids -dbtype prot # make the database

Blasting against Swissprot database

blastx -query ESA_reference_09_20_16.fasta -db uniprot_sprot.pep -num_threads 12 -max_target_seqs 1 -outfmt 6 -out ESA_SwPr_BlstX.outfmt6

blastp -query ESA_ref_092016.pep -db uniprot_sprot.pep -num_threads 12 -max_target_seqs 1 -outfmt 6 -out ESA_SwPr_BlstP.outfmt6

Running blast against Uniref90 on Sep 28 2016

blastx -query ESA_reference_09_20_16.fasta -db uniprot_uniref90.trinotate_v2.0.pep -num_threads 12 -max_target_seqs 1 -outfmt 6 -out ESA_Uniref90_BlstX.outfmt6

blastp -query ESA_ref_092016.pep -db uniprot_uniref90.trinotate_v2.0.pep -num_threads 12 -max_target_seqs 1 -outfmt 6 -out ESA_Uniref90_BlastP.outfmt6

Running HMMER to identify protein domains

hmmscan --cpu 12 --domtblout ESA_PFAM.out Pfam-A.hmm ESA_ref_092016.pep >

ESA_PFAM.log

Running SignalP

signalp -f short -n ESA_signalP.out ESA_ref_092016.pep

Running tmHMM

tmhmm --short ESA_ref_092016.pep > ESA_tmhmm.out

Running transdecoder
TransDecoder.LongOrfs -t ESA_reference.fasta

Making the gene trans map for Trinotate

/opt/trinity/2.0.6/gcc/util/support_scripts/get_Trinity_gene_to_trans_map.pl
ESA_reference_09_20_16.fasta >> ESA_reference_09_20_16.fasta.gene_trans_map

Running Trinotate

Trinotate Trinotate.sprot_uniref90.20150131.boilerplate.sqlite init --gene_trans_map
ESA_reference_09_20_16.fasta.gene_trans_map --transcript_fasta
ESA_reference_09_20_16.fasta --transdecoder_pep ESA_ref_092016.pep

Trinotate Trinotate.sprot_uniref90.20150131.boilerplate.sqlite LOAD_swissprot_blastp
ESA_SwPr_BlstP.outfmt6
Trinotate Trinotate.sprot_uniref90.20150131.boilerplate.sqlite LOAD_trembl_blastp
ESA_Uniref90_BlastP.outfmt6
Trinotate Trinotate.sprot_uniref90.20150131.boilerplate.sqlite LOAD_pfam ESA_PFAM.out
Trinotate Trinotate.sprot_uniref90.20150131.boilerplate.sqlite LOAD_tmhmm
ESA_tmhmm.out
Trinotate Trinotate.sprot_uniref90.20150131.boilerplate.sqlite LOAD_signalp ESA_signalP.out

Trinotate Trinotate.sprot_uniref90.20150131.boilerplate.sqlite LOAD_swissprot_blastx
ESA_SwPr_BlstX.outfmt6
Trinotate Trinotate.sprot_uniref90.20150131.boilerplate.sqlite LOAD_trembl_blastx
ESA_Uniref90_BlstX.outfmt6

Trinotate Trinotate.sprot_uniref90.20150131.boilerplate.sqlite report -E 1e-5 --pfam_cutoff
DNC > ESA_annotation_report_31Oct2016.xls

RUVg, Filtering and EdgeR

```
source("https://bioconductor.org/biocLite.R")
biocLite("RUVSeq")
biocLite("zebrafishRNASeq")
biocLite("edgeR")

library(edgeR)
library(RUVSeq)

### Filtering the data ###

setwd("~/Google Drive/PDF/NGS/ESA_Bowtie2_xprs/RUVSeq")
Ruvg_count_matrix <- read.csv("~/Google
Drive/PDF/NGS/ESA_Bowtie2_xprs/RUVSeq/Ruvg_count_matrix.csv", row.names=1)
View(Ruvg_count_matrix)
keep <- rowSums(cpm(Ruvg_count_matrix)>1) >= 3
Ruvg_count_matrix <- Ruvg_count_matrix[keep,]
filter <- apply(Ruvg_count_matrix, 1, function(x) length(x[x>5])>=2)
# arranging the file for further analysis
filtered <- Ruvg_count_matrix[filter,]
write.csv(filtered,file="~/Google
Drive/PDF/NGS/ESA_Bowtie2_xprs/RUVSeq/EdgeRRUVgcount_matrix.csv")

## try http:// if https:// URLs are not supported
source("https://bioconductor.org/biocLite.R")
biocLite("RUVSeq")
biocLite("zebrafishRNASeq")
biocLite("edgeR")

library(edgeR)
library(RUVSeq)

### Filter the data ########
setwd("~/Google Drive/PDF/NGS/ESA_Bowtie2_xprs/RUVSeq")
Ruvg_count_matrix <- read.csv("~/Google
Drive/PDF/NGS/ESA_Bowtie2_xprs/RUVSeq/Ruvg_count_matrix.csv", row.names=1)
View(Ruvg_count_matrix)
keep <- rowSums(cpm(Ruvg_count_matrix)>1) >= 3
Ruvg_count_matrix <- Ruvg_count_matrix[keep,]
head(Ruvg_count_matrix) #shows the first few lines of the dataset
tail(Ruvg_count_matrix) # shows the last few lines of the dataset

### read in data from csv file
### while importing the database select heading=Yes and Rownames=Use first column
filter <- apply(Ruvg_count_matrix, 1, function(x) length(x[x>5])>=2)
```

```
# arranging the file for further analysis
filtered <- Ruvg_count_matrix[filter,] # puts the filtered dataset in the filtered filename for
further use
write.csv(filtered,file="~/Google
Drive/PDF/NGS/ESA_Bowtie2_xprs/RUVSeq/EdgeRRUVgcount_matrix.csv")
genes <- rownames(filtered)[grep("c", rownames(filtered))] #tells the number of transcripts in
the filtered data without the ERCC spikeins

spikes <- rownames(filtered)[grep("ERCC", rownames(filtered))] #tells you the number of
transcripts for ERCC spikeins in the filtered data

#x <- as.factor(rep(c("DA","DC","DG","IN","RA","RC","RG"), each=3)) # picks out the rows
defined according to the experimental and control
groups = c("DA","DA", "DA", "DC","DC" ,"DC", "DG","DG", "DG", "IN",
"IN","RA","RA","RA", "RC", "RC", "RC","RG", "RG","RG") # picks out the rows defined

x <- as.factor (groups)
x
set <- newSeqExpressionSet(as.matrix(filtered),
                     phenoData = data.frame(x, row.names=colnames(filtered)))
set

library(RColorBrewer)
colors <- brewer.pal(7, "Set2")
tiff ("no_norm_Final.tiff", height = 6, width = 8,
    units = 'in', res = 1000, compression = 'lzw')
plotRLE(set, outline=FALSE, ylim=c(-8, 8), col=colors[x])
dev.off()
tiff ("no_norm_Final.tiff", height = 6, width = 8,
    units = 'in', res = 1000, compression = 'lzw')
plotPCA(set, col=colors[x], cex=1)
dev.off()


set1 <- betweenLaneNormalization(set, which="upper")
tiff ("norm2_Final.tiff", height = 6, width = 8,
    units = 'in', res = 1000, compression = 'lzw')
plotRLE(set1, outline=FALSE, ylim=c(-8, 8), col=colors[x])
dev.off()
tiff ("norm2_final.tiff", height = 6, width = 8,
    units = 'in', res = 1000, compression = 'lzw')
plotPCA(set1, col=colors[x], cex=1)
dev.off()
```

```
set2 <- RUVg(set1, spikes, k=1)
pData(set2)
tiff ("ERCC_Norm2_Final.tiff", height = 6, width = 8,
    units = 'in', res = 1000, compression = 'lzw')
plotRLE(set2, outline=FALSE, ylim=c(-8, 8), col=colors[x])
dev.off()
tiff ("ERCC_Norm2_Final.tiff", height = 6, width = 8,
    units = 'in', res = 1000, compression = 'lzw')
plotPCA(set2, cex=1, col=colors[x])
dev.off()

# saving the set2 file from RUVg as a .csv for further EdgeR analysis
write.csv(set2@assayData$normalizedCounts, "~/Google
Drive/PDF/NGS/Trinotate/EdgeR/FilteredNormalizedRUVg_ESA.csv")


##########################
### EdgeR
##########################

biocLite("NBPSeq")
biocLite("edgeR")
library(NBPSeq)
library(edgeR)
setwd("~/Google Drive/PDF/NGS/Trinotate/EdgeR")
CountMatrix <- read.csv("~/Google
Drive/PDF/NGS/Trinotate/EdgeR/FilteredNormalizedRUVg_ESA.csv",
stringsAsFactors=FALSE)
targets <- read.csv("~/Google Drive/PDF/NGS/Trinotate/EdgeR/GroupESA_EdgeR.csv")
Group <- factor(paste(targets$Treat,targets$Time,sep="."))
cbind(targets,Group=Group)
design <- model.matrix(~0+Group)
colnames(design) <- levels(Group)

### Making the count matrix with treatment and time
### IN corresponds to I (intact/intermolt)


ModifiedCountMatrix <- DGEList(counts=CountMatrix[,2:21],
group=Group,genes=CountMatrix[,1:2])
ModifiedCountMatrix
DispersedModifiedCountMatrix <- estimateDisp(ModifiedCountMatrix, design)
fit <- glmFit(DispersedModifiedCountMatrix, design)
my.contrasts <- makeContrasts(DMSO.1dvsDMSO.3d = (DMSO.1d-IN.0d)-(DMSO.3d-IN.0d),
```

```
                    DMSO.3dvsDMSO.7d = (DMSO.3d-IN.0d)-(DMSO.7d-IN.0d),
                    DMSO.1dvsDMSO.7d = (DMSO.1d-IN.0d)-(DMSO.7d-IN.0d),
                    Rapamycin.1dvsRapamycin.3d = (Rapamycin.1d-IN.0d)-(Rapamycin.3d-
IN.0d),
                    Rapamycin.1dvsRapamycin.7d = (Rapamycin.1d-IN.0d)-(Rapamycin.7d-
IN.0d),
                    Rapamycin.3dvsRapamycin.7d = (Rapamycin.3d-IN.0d)-(Rapamycin.7d-
IN.0d),
                    DMSO.1dvsRapamycin.1d = (DMSO.1d-IN.0d)-(Rapamycin.1d-IN.0d),
                    DMSO.3dvsRapamycin.3d = (DMSO.3d-IN.0d)-(Rapamycin.3d-IN.0d),
                    DMSO.7dvsRapamycin.7d = (DMSO.7d-IN.0d)-(Rapamycin.7d-IN.0d),
                    levels=design)


lrt.DAvsRA <- glmLRT(fit, contrast=my.contrasts[,"DMSO.1dvsRapamycin.1d"])
topTags(lrt.DAvsRA)
ArrangedlrtDAvsRA <- topTags(lrt.DAvsRA, n=35696)
Gene <- ArrangedlrtDAvsRA[[1]]$X
log2FoldChange <- ArrangedlrtDAvsRA[[1]]$logFC
pvalue <- ArrangedlrtDAvsRA[[1]]$PValue
padj <- ArrangedlrtDAvsRA[[1]]$FDR
res <- data.frame(Gene,log2FoldChange,pvalue,padj)
significantresDAvsRA<-subset(res,padj<0.05)
DiffExpGenesDAvsRA<-significantresDAvsRA$Gene

lrt.DCvsRC <- glmLRT(fit, contrast=my.contrasts[,"DMSO.3dvsRapamycin.3d"])
topTags(lrt.DCvsRC)
ArrangedlrtDCvsRC <- topTags(lrt.DCvsRC, n=35696)
Gene <- ArrangedlrtDCvsRC[[1]]$X
log2FoldChange <- ArrangedlrtDCvsRC[[1]]$logFC
pvalue <- ArrangedlrtDCvsRC[[1]]$PValue
padj <- ArrangedlrtDCvsRC[[1]]$FDR
res <- data.frame(Gene,log2FoldChange,pvalue,padj)
significantresDCvsRC<-subset(res,padj<0.05)
DiffExpGenesDCvsRC<-significantresDCvsRC$Gene

lrt.DGvsRG <- glmLRT(fit, contrast=my.contrasts[,"DMSO.7dvsRapamycin.7d"])
topTags(lrt.DGvsRG)
ArrangedlrtDGvsRG <- topTags(lrt.DGvsRG, n=35696)
Gene <- ArrangedlrtDGvsRG[[1]]$X
log2FoldChange <- ArrangedlrtDGvsRG[[1]]$logFC
pvalue <- ArrangedlrtDGvsRG[[1]]$PValue
padj <- ArrangedlrtDGvsRG[[1]]$FDR
```

```
res <- data.frame(Gene,log2FoldChange,pvalue,padj)
significantresDGvsRG<-subset(res,padj<0.05)
DiffExpGenesDGvsRG<-significantresDGvsRG$Gene

lrt.DAvsDC <- glmLRT(fit, contrast=my.contrasts[,"DMSO.1dvsDMSO.3d"])
topTags(lrt.DAvsDC)
ArrangedlrtDAvsDC <- topTags(lrt.DAvsDC, n=35696)
Gene <- ArrangedlrtDAvsDC[[1]]$X
log2FoldChange <- ArrangedlrtDAvsDC[[1]]$logFC
pvalue <- ArrangedlrtDAvsDC[[1]]$PValue
padj <- ArrangedlrtDAvsDC[[1]]$FDR
res <- data.frame(Gene,log2FoldChange,pvalue,padj)
significantresDAvsDC<-subset(res,padj<0.05)
DiffExpGenesDAvsDC<-significantresDAvsDC$Gene

lrt.DAvsDG <- glmLRT(fit, contrast=my.contrasts[,"DMSO.1dvsDMSO.7d"])
topTags(lrt.DAvsDG)
ArrangedlrtDAvsDG <- topTags(lrt.DAvsDG, n=35696)
ArrangedlrtDAvsDG
Gene <- ArrangedlrtDAvsDG[[1]]$X
log2FoldChange <- ArrangedlrtDAvsDG[[1]]$logFC
pvalue <- ArrangedlrtDAvsDG[[1]]$PValue
padj <- ArrangedlrtDAvsDG[[1]]$FDR
res <- data.frame(Gene,log2FoldChange,pvalue,padj)
significantresDAvsDG<-subset(res,padj<0.05)
DiffExpGenesDAvsDG<-significantresDAvsDG$Gene

lrt.DCvsDG <- glmLRT(fit, contrast=my.contrasts[,"DMSO.3dvsDMSO.7d"])
topTags(lrt.DCvsDG)
ArrangedlrtDCvsDG <- topTags(lrt.DCvsDG, n=35696)
Gene <- ArrangedlrtDAvsDC[[1]]$X
log2FoldChange <- ArrangedlrtDCvsDG[[1]]$logFC
pvalue <- ArrangedlrtDCvsDG[[1]]$PValue
padj <- ArrangedlrtDCvsDG[[1]]$FDR
res <- data.frame(Gene,log2FoldChange,pvalue,padj)
significantresDCvsDG<-subset(res,padj<0.05)
DiffExpGenesDCvsDG<-significantresDCvsDG$Gene



lrt.RAvsRC <- glmLRT(fit, contrast=my.contrasts[,"Rapamycin.1dvsRapamycin.3d"])
topTags(lrt.RAvsRC)
ArrangedlrtRAvsRC <- topTags(lrt.RAvsRC, n=35696)
Gene <- ArrangedlrtRAvsRC[[1]]$X
```

```
log2FoldChange <- ArrangedlrtRAvsRC[[1]]$logFC
pvalue <- ArrangedlrtRAvsRC[[1]]$PValue
padj <- ArrangedlrtRAvsRC[[1]]$FDR
res <- data.frame(Gene,log2FoldChange,pvalue,padj)
significantresRAvsRC<-subset(res,padj<0.05)
DiffExpGenesRAvsRC<-significantresRAvsRC$Gene


lrt.RAvsRG <- glmLRT(fit, contrast=my.contrasts[,"Rapamycin.1dvsRapamycin.7d"])
topTags(lrt.RAvsRG)
ArrangedlrtRAvsRG <- topTags(lrt.RAvsRG, n=35696)
Gene <- ArrangedlrtRAvsRG[[1]]$X
log2FoldChange <- ArrangedlrtRAvsRG[[1]]$logFC
pvalue <- ArrangedlrtRAvsRG[[1]]$PValue
padj <- ArrangedlrtRAvsRG[[1]]$FDR
res <- data.frame(Gene,log2FoldChange,pvalue,padj)
significantresRAvsRG<-subset(res,padj<0.05)
DiffExpGenesRAvsRG<-significantresRAvsRG$Gene


lrt.RCvsRG <- glmLRT(fit, contrast=my.contrasts[,"Rapamycin.3dvsRapamycin.7d"])
topTags(lrt.RCvsRG)
ArrangedlrtRCvsRG <- topTags(lrt.RCvsRG, n=35696)
Gene <- ArrangedlrtRCvsRG[[1]]$X
log2FoldChange <- ArrangedlrtRCvsRG[[1]]$logFC
pvalue <- ArrangedlrtRCvsRG[[1]]$PValue
padj <- ArrangedlrtRCvsRG[[1]]$FDR
res <- data.frame(Gene,log2FoldChange,pvalue,padj)
significantresRCvsRG<-subset(res,padj<0.05)
DiffExpGenesRCvsRG<-significantresRCvsRG$Gene

### Gives you the differentially expressed genes in the whole dataset ###

DiffExpGenesAll<-
c(as.character(DiffExpGenesRAvsRC),as.character(DiffExpGenesRAvsRG),as.character(DiffE
xpGenesRCvsRG),as.character(DiffExpGenesDAvsDC),as.character(DiffExpGenesDAvsDG),a
s.character(DiffExpGenesDCvsDG),as.character(DiffExpGenesDAvsRA),as.character(DiffExp
GenesDCvsRC),as.character(DiffExpGenesDGvsRG))

DiffExpGenesAll<-data.frame(unique(DiffExpGenesAll))
head(DiffExpGenesAll)
write.csv(DiffExpGenesAll,file="~/Google
Drive/PDF/NGS/Trinotate/EdgeR/DiffExpGenesAll.csv")
```

```
# Make a basic volcano plot
with(res, plot(log2FoldChange, -log10(pvalue), col = "grey", pch=20, main="DMSO-Day7 vs
Rapamycin-Day7", xlim=c(-12,12),ylim=c(0,12)))
# Add colored points: red if padj<0.5, orange of log2FC>1, green if both)
with(subset(res, padj<.50  & log2FoldChange>3), points(log2FoldChange, -log10(pvalue),
pch=20, col="green"))
with(subset(res, padj<.50  & log2FoldChange<(-3)), points(log2FoldChange, -log10(pvalue),
pch=20, col="red"))
```
